# Additive engineering for Sb_2_S_3_ indoor photovoltaics with efficiency exceeding 17%

**DOI:** 10.1038/s41377-024-01620-0

**Published:** 2024-10-02

**Authors:** Xiao Chen, Xiaoxuan Shu, Jiacheng Zhou, Lei Wan, Peng Xiao, Yuchen Fu, Junzhi Ye, Yi-Teng Huang, Bin Yan, Dingjiang Xue, Tao Chen, Jiejie Chen, Robert L. Z. Hoye, Ru Zhou

**Affiliations:** 1 School of Electrical Engineering and Automation, Hefei University of Technology, Hefei, 230009 PR China; 2 Department of Environmental Science and Engineering, Key Laboratory of Urban Pollutant Conversion, University of Science and Technology of China, Hefei, 230009 PR China; 3 Hefei National Research Center for Physical Sciences at the Microscale, School of Chemistry and Materials Science, University of Science and Technology of China, Hefei, 230026 PR China; 4 Department of Chemistry, Inorganic Chemistry Laboratory, University of Oxford, South Parks Road, Oxford, OX1 3QR UK; 5 Beijing National Laboratory for Molecular Sciences, CAS Key Laboratory of Molecular Nanostructure and Nanotechnology, Institute of Chemistry, Chinese Academy of Sciences, Beijing, 100190 PR China

**Keywords:** Photonic devices, Optoelectronic devices and components

## Abstract

Indoor photovoltaics (IPVs) have attracted increasing attention for sustainably powering Internet of Things (IoT) electronics. Sb_2_S_3_ is a promising IPV candidate material with a bandgap of ~1.75 eV, which is near the optimal value for indoor energy harvesting. However, the performance of Sb_2_S_3_ solar cells is limited by nonradiative recombination, which is dependent on the quality of the absorber films. Additive engineering is an effective strategy to fine tune the properties of solution-processed films. This work shows that the addition of monoethanolamine (MEA) into the precursor solution allows the nucleation and growth of Sb_2_S_3_ films to be controlled, enabling the deposition of high-quality Sb_2_S_3_ absorbers with reduced grain boundary density, optimized band positions, and increased carrier concentration. Complemented with computations, it is revealed that the incorporation of MEA leads to a more efficient and energetically favorable deposition for enhanced heterogeneous nucleation on the substrate, which increases the grain size and accelerates the deposition rate of Sb_2_S_3_ films. Due to suppressed carrier recombination and improved charge-carrier transport in Sb_2_S_3_ absorber films, the MEA-modulated Sb_2_S_3_ solar cell yields a power conversion efficiency (PCE) of 7.22% under AM1.5 G illumination, and an IPV PCE of 17.55% under 1000 lux white light emitting diode (WLED) illumination, which is the highest yet reported for Sb_2_S_3_ IPVs. Furthermore, we construct high performance large-area Sb_2_S_3_ IPV minimodules to power IoT wireless sensors, and realize the long-term continuous recording of environmental parameters under WLED illumination in an office. This work highlights the great prospect of Sb_2_S_3_ photovoltaics for indoor energy harvesting.

## Abstract (web summary)

We demonstrate an effective additive engineering strategy to construct Sb_2_S_3_ indoor photovoltaics (IPVs) with 17.55% efficiency under 1000 lux WLED illumination, enabling high-performance IPV minimodules for powering IoT wireless electronics.

## Graphical abstract

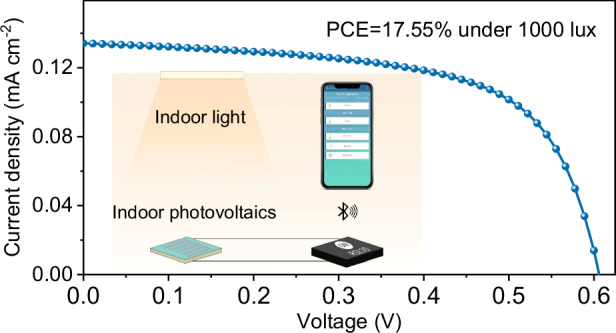

## Introduction

The rapid development of Internet of Things (IoT) technologies is leading to an ongoing exponentially growing market of smart devices^1^. Such end nodes are usually designed for cyclic operation and low power consumption (microwatts to milliwatts), and having a reliable and sustainable long-term power supply is critical for the success of IoT technologies. Currently, autonomous IoT nodes are mostly powered using batteries. However, the short lifespan of batteries not only limits the power consumption and size of IoT devices, but also restricts the applications to the cases which are compatible with battery replacement and maintenance. Solely relying on batteries to power IoT devices might not sustain the rapidly growing size of the IoT ecosystem as it proceeds to a trillion nodes, where it is predicted that >100 billion batteries will have to be disposed of or replaced each year^2^. In this regard, integrated energy systems for harvesting ambient energy (e.g., indoor lighting, mechanical energy, or thermal energy) are now expected to serve as alternatives, or work complementarily to batteries^1,3^. Indoor photovoltaics (IPVs), which capture energy from ambient lighting (either from artificial light sources, or daylight), have significant potential to provide sustainable power for driving wireless IoT nodes that communicate using a range of protocols, such as Bluetooth low energy, RFID tags, LoRa, passive Wi-Fi, Zigbee, ANT, etc^4^. Indeed, IPVs are deployable in view of their reliance on radiative energy transfer and indoor lighting being ubiquitously available and predictable. Furthermore, IPVs afford relatively high energy density compared to other ambient energy harvesting technologies^2^. Therefore, the development of high performance IPVs is important for sustainable IoT applications^5^. Indoor illuminances typically range from 200 to 500 lux for private households, and 500–1500 lux for office, commercial and industrial areas. For commonly used cold white light emitting diodes (WLEDs), an illuminance of 500–1000 lux (0.14–0.28 mW cm^−2^) is 300–700 times lower compared to “1-sun” (AM1.5 G, 100 mW cm^−2^)^6^. The narrow emission spectra of indoor light sources (e.g., LEDs and fluorescent lamps (FLs)) range from 400 to 700 nm, which determines the optimal bandgap for indoor light-absorbing materials to be around 1.80–2.00 eV^2,7,8^. Therefore, crystalline silicon (c-Si), which dominates the outdoor PVs market, would not be well suited for indoor energy harvesting, due to its small bandgap (~1.12 eV), as well as high dark current densities, which together limit its performance under the lower irradiances from indoor lighting^9^. The state-of-the-art commercial solution up to now for IPVs is hydrogenated amorphous silicon (a-Si:H), with a bandgap of 1.7–2.0 eV. The PCEs of commercially available standard a-Si:H module devices typically range from 4.4% to 9.2%^2^. To date, emerging next-generation solar cells, including organic photovoltaics (OPVs), dye-sensitized solar cells (DSSCs), and lead-halide perovskite solar cells (LHPSCs) have also been investigated for IPV applications^5,10–14^. However, DSSCs and OPVs are limited by the use of toxic solvents and expensive small molecules; LHPSCs involve the use of high contents of lead in the thin film absorber, which exceeds the limits of the EU restriction of hazardous substances directive (RoHS), and this may act as a barrier to commercialization. Efficient IPVs comprised of stable materials that can be simply processed and are comprised of RoHS-compliant elements are missing.

Sb_2_S_3_ is an earth-abundant, low-toxicity, and stable material with a bandgap of ~1.75 eV, close to the optimal bandgap value for IPV applications^7^. Moreover, Sb_2_S_3_ possesses excellent materials and optoelectronic properties, including simple binary composition, quasi-one-dimensional crystal structure, high absorption coefficients (10^4^–10^5 ^cm^−1^), low melting points (~500 °C) and high vapor pressures. These merits enable the low-temperature fabrication of high performance, flexible, and lightweight Sb_2_S_3_ devices for powering IoT electronics. According to calculations by Hoye et al., the spectroscopic limited maximum efficiency reaches 47% under 1000 lux white LED lighting^2^. However, up to now, the development of Sb_2_S_3_ solar cells has mainly focused on its performance under 1-sun illumination (i.e., outdoor photovoltaics). Since the illumination intensity and emission spectra of indoor light sources in most building and office environments, such as FLs and LEDs, are strikingly different from 1-sun conditions, it is necessary to carefully study the IPV performance of Sb_2_S_3_ photovoltaics^3,15^. As far as we know, only Zheng et al. reported the IPV performance for Sb_2_S_3_ devices, which delivered 16.37% indoor efficiency when illuminated with a 1000 lux WLED^16^. The application of Sb_2_S_3_ devices for powering IoT electronics has, to our knowledge, never been demonstrated before.

Thanks to endeavors from a growing community of researchers focusing on this chalcogenide photovoltaic materials system, great achievements have been made for Sb_2_S_3_ solar cells. To date, the mesoporous and planar Sb_2_S_3_ photovoltaics have delivered record efficiencies of 7.5% and 8.0% under 1-sun illumination, respectively^17,18^. However, such efficiency values still lag far behind the theoretical maximum efficiency of 28.64% according to the Shockley-Queisser limit^19^. The severe charge-carrier recombination at grain boundaries (GBs) and interfaces is one of the critical problems that limits the performance of Sb_2_S_3_ solar cells^19,20^. Over the past decade, researchers have explored a variety of approaches to enhance the efficiency of Sb_2_S_3_ solar cells, such as ion doping^19,21^, additive engineering^16,22^, surface passivation^23^, configuration optimization^24^, etc. Additive engineering has attracted tremendous attention because it is a simple strategy to regulate the deposition processes as well as the film properties for solution-processed Sb_2_S_3_ films, such as deposition rate, growth orientation, film morphology, etc^18^. To date, a number of additives, including sodium dodecyl sulfate^16^, thioacetamide^18^, tartaric acid^25^, tetramethylammonium hexafluoro phosphonate^22^, and ammonium sulfide^26^ have been investigated to adjust the chemical bath deposition or hydrothermal deposition processes of Sb_2_S_3_ films. For instance, Wang et al. used thioacetamide together with sodium thiosulfate to act as dual sulfur sources and achieved noticeable improvements in the device efficiency due to the advantageous hydrolysis cooperation between two sulfur sources^18^. Han et al. employed tetramethylammonium hexafluoro phosphonate that could coordinate with Sb^3+^ due to multidentate anchoring, and improved the morphology of Sb_2_S_3_ films and reduced the trap state density^22^. Therefore, additive engineering is promising for optimizing film properties and enhancing the photovoltaic performance of Sb_2_S_3_ solar cells.

As a complexing agent that has a hydrophilic hydroxyl group (-OH) and amino group (-NH_2_), monoethanolamine (MEA) is expected to be capable of effectively regulating the nucleation and growth environment for film deposition^27,28^. In this work, we obtained high-quality Sb_2_S_3_ films through an additive-modulated hydrothermal deposition method by incorporating a small amount of MEA into the precursor solution. This additive engineering strategy increases the grain size and accelerates the deposition rate of these absorbers, which further improve the optoelectronic properties of absorber films. The MEA-modulated planar Sb_2_S_3_ solar cells delivered a PCE of 7.22% under 1-sun illumination and a remarkable indoor PCE of 17.55% under 1000 lux white LED (WLED) illumination. We further demonstrated the use of large-area Sb_2_S_3_ minimodules to power low-power portable electronics.

## Results

### Deposition of high-quality Sb_2_S_3_ films

**Fig. 1:**
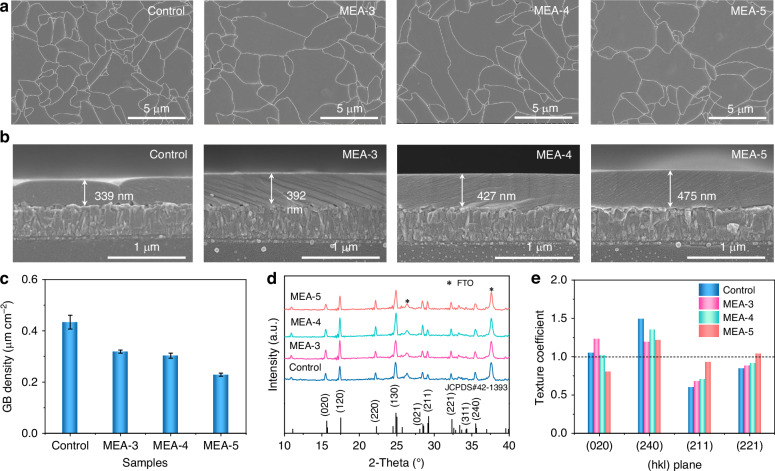
**Morphological and structural properties of Sb**_**2**_**S**_**3**_ **films**. **a**, **b** Top-view and cross-sectional SEM images and (**c**) GB densities of hydrothermally deposited Sb_2_S_3_ thin films prepared without (control) and with MEA additives. Three samples were measured to determine the mean GB density values shown, and the error bars represent the standard deviation. **d**, **e** XRD patterns and texture coefficients of the dominant (020), (240), (211), and (221) peaks of the control film and MEA-Sb_2_S_3_ films prepared with different concentrations of MEA additives

In this work, we employed a hydrothermal deposition method to grow Sb_2_S_3_ films onto FTO/SnO_2_/CdS substrates. Antimony potassium tartrate (APT) and sodium thiosulfate were used as the antimony and sulfur sources, respectively. A small amount of MEA was used as an additive in the precursor solution to regulate the deposition of Sb_2_S_3_ films. Figure 1a shows top-view scanning electron microscopy (SEM) images of as-deposited Sb_2_S_3_ thin films prepared without the additive (control), and with different concentrations of MEA additives. For convenience, the Sb_2_S_3_ films obtained with MEA concentrations of 3 μg mL^−1^, 4 μg mL^−1^, and 5 μg mL^−1^ in the hydrothermal deposition are labeled as MEA-3, MEA-4, and MEA-5, respectively. As shown, the control sample displays grains smaller than 5 μm, consistent with that reported for hydrothermally-deposited Sb_2_S_3_ films^19^. It is worth noting that we have previously established that SEM images are suitable for evaluating the grain size of Sb_2_S_3_ films^19^. With the addition of MEA, the grain size of Sb_2_S_3_ films involves a significant increase, and some Sb_2_S_3_ grains exceed 10 μm with an MEA concentration of 4 μg mL^−1^. The increase in grain size is accompanied by a decrease in GB density, which can be defined as the GB length per unit area. As shown in Fig. 1c, the GB density of Sb_2_S_3_ films decreases from 0.434 ± 0.027 μm cm^−2^ for the control sample to 0.319 ± 0.006, 0.303 ± 0.010, and 0.229 ± 0.006 μm cm^−2^ for the MEA-3, MEA-4, and MEA-5 samples, respectively. The reduced GB density of the absorber films is expected to favor the suppression of non-radiative recombination^29^. The cross-sectional SEM images (Fig. 1b) reveal that the Sb_2_S_3_ films have a gradual increase in thickness with the addition of MEA. The film thickness increased from 328 ± 11 nm for the control sample to 399 ± 8 nm, 428 ± 6 nm, and 480 ± 5 nm for the MEA-3, MEA-4, and MEA-5 samples, respectively. This implies that the addition of MEA to the precursor solution accelerates the deposition rate of Sb_2_S_3_ films. The 2D and 3D morphology spatial maps from atomic force microscopy (AFM) measurements (Supplementary Fig. S1) further indicate that the MEA-4 film samples show larger grains compared to the control. Moreover, the addition of MEA also leads to a reduction in the root-mean-square roughness of absorber films from 18.4 nm (the control sample) to 16.2 nm (the MEA-4 sample). The flatter Sb_2_S_3_ films should be beneficial for the formation of high-quality heterojunctions between the absorber layer and the solution-processed hole transport layer (HTL) on top.

X-ray diffraction (XRD) patterns of Sb_2_S_3_ films (Fig. 1d) show that the diffraction peaks can be indexed to pure orthorhombic Sb_2_S_3_ (JCPDS #42-1393), with the background peaks from FTO substrates as marked by the asterisks^20^. The diffraction peaks of Sb_2_S_3_ films reveal negligible shifts with the addition of MEA, indicating that the MEA additive could not be incorporated into the Sb_2_S_3_ lattice. Compared to the control film, the MEA-Sb_2_S_3_ films exhibit slightly reduced intensities for (020) and (240) peaks as well as enhanced intensities for (211) and (221) peaks. The texture coefficients (TC) of (020), (240), (211) and (221) planes of Sb_2_S_3_ films are further calculated to evaluate the changes in the film orientation, as shown in Fig. 1e. It can be seen that the addition of MEA results in the [211] and [221] preferred orientations for Sb_2_S_3_ films. As we know, Sb_2_S_3_ has a quasi-1D crystal structure consisting of numerous parallel (Sb_4_S_6_)_*n*_ ribbons with van der Waals interactions between them^20^. Hence the carrier transport along (Sb_4_S_6_)_*n*_ ribbons is much more efficient than the charge hopping between ribbons, and the enhanced [hk1] preferred orientation should be favorable for charge-carrier transport in the vertically-structured devices.

Conductive AFM (c-AFM) characterization of the control and MEA-4 films (Supplementary Fig. S2a, b) reveal that the MEA-4 samples display higher current on the film surface than the control sample. The surface current intensity distributions (Supplementary Fig. S2c, d) show that both samples have obvious current fluctuations at GBs, which might be caused by the reduction in the carrier density at GBs^19^. Compared to the control sample, the MEA-4 films show reduced current fluctuations and increased average current intensity. This should be associated with the decrease of GBs for the MEA-4 sample. The alleviated current fluctuation on the absorber films would facilitate the collection of charge-carriers. To further evaluate the conductivity change of Sb_2_S_3_ films, we prepared devices with the simple device structure of FTO/Sb_2_S_3_/Au and measured *I–V* curves in the dark (Supplementary Fig. S3). The conductivity of films can be calculated by the equation^24^: $I={\sigma }_{0}A{L}^{-1}V$, where ${\sigma }_{0}$ is the conductivity, $A$ represents the area of the apparatus (0.06 cm^2^), $L$ is the thickness of Sb_2_S_3_ layer (here the thicknesses of the control and MEA-4 films are 339 nm and 427 nm, respectively), and *I* and *V* are the current and the voltage, respectively. The estimated conductivities of the control and MEA-4 films are 3.55 × 10^−5^ and 5.96 × 10^−5^ S cm^−1^, respectively. The increase in the conductivity of MEA-4 film might be associated to the increase in grain size, consistent with the c-AFM results^30^.

### Mechanisms of additive engineering

We now investigate the underlying mechanism for the impact of the MEA additives on the film properties of Sb_2_S_3_. First, we hypothesize that the MEA additive is absent in as-deposited Sb_2_S_3_ films, since the decomposition temperature of MEA molecules (~200 °C) is much lower than the post-annealing temperature for Sb_2_S_3_ films (~370 °C)^31^. The X-ray photoelectron spectroscopy (XPS) and Raman characterization of the control and MEA-4 films were performed to test this hypothesis. XPS spectra (Supplementary Fig. S4) and Raman spectra (Supplementary Fig. S5) reveal that both the control and MEA-4 film samples share nearly identical characteristic peaks, indicating that the incorporation of MEA additives exerts no impact on the elemental composition and chemical states of Sb_2_S_3_ films. Hence, we speculate that MEA only plays roles in the hydrothermal deposition process of Sb_2_S_3_ absorber films.

**Fig. 2:**
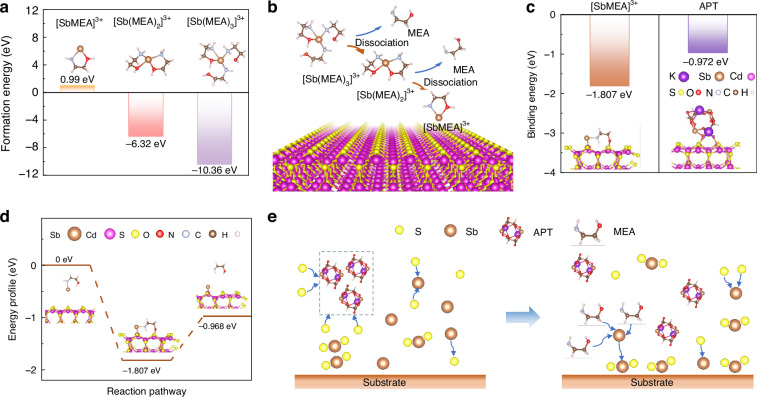
**Mechanisms of additive engineering for the deposition of Sb**_**2**_**S**_**3**_ **films**. **a** The formation energy of [Sb(MEA)_*n*_]^3+^, *n* = 1, 2, and 3. **b** Schematic diagram depicting the sequential dissociation of [Sb(MEA)_3_]^3+^ into [Sb(MEA)_2_]^3+^ and further into [SbMEA]^3+^ on the CdS substrate with the addition of MEA. **c** The binding energy between the Sb^3+^ complexes ([SbMEA]^3+^ and APT) and the CdS substrate. **d** The reaction energy profile diagram of [SbMEA]^3+^ binding to the CdS substrate and releasing the MEA ligand. **e** Schematic illustration of the evolution of the Sb_2_S_3_ deposition process on the substrate with the addition of MEA

In this study, MEA as the complexing agent was incorporated into the precursor solution to regulate the deposition process. MEA can be uniformly dissolved into the precursor solution. To investigate the possible mechanisms of additive engineering, density functional theory (DFT) calculations were employed to examine the role of ligands and their interactions with the substrate. Firstly, DFT calculations reveal that the simultaneous coordination of the O and N atoms from MEA to Sb^3+^ gives the lowest formation energy compared to single-atom coordination scenarios (Supplementary Fig. S6 and Supplementary Table S1), resulting in the formation of the most favorable configuration, which could enhance the stability of the complex. Then, the models of [Sb(MEA)_*n*_]^3+^ complexes were constructed with different coordination numbers, where *n* = 1, 2, and 3. Coordination numbers exceeding 3 were excluded from consideration due to significant steric hindrance among MEA molecules. As shown in Fig. 2a, [Sb(MEA)_3_]^3+^ manifests the lowest formation energy, thereby signifying it as the most stable coordination state (Supplementary Tables S2 and S3). Moreover, the dissociation process of [Sb(MEA)_3_]^3+^ into [Sb(MEA)_2_]^3+^ and subsequently into [Sb(MEA)]^3+^ necessitates an external energy input of merely 1.003 eV complex^−1^ and 0.943 eV complex^−1^, respectively (Fig. 2b, Supplementary Fig. S7 and Supplementary Table S4). This indicates that continuous thermal energy input into the system allows the coexistence of the three forms of MEA-complexed Sb^3+^ during the hydrothermal deposition stage. Ultimately, Sb^3+^ might bind to the CdS substrate in the form of [SbMEA]^3+^, owing to the complete exposure of Sb^3+^ in this configuration. This specific configuration of the ligand is advantageous for the deposition and growth of Sb_2_S_3_ on the substrate. In the hydrothermal deposition of Sb_2_S_3_ films, Sb^3+^ ions were introduced in the form of C_8_H_4_K_2_O_12_Sb_2_, necessitating an examination of the binding capacities of this specific coordination complex at the interface. The strong chelating effect of the tartrate ions introduces complexity to the binding process of Sb^3+^ to the substrate, thereby impeding their effective stabilization on the substrate surface^30^. Moreover, the considerable steric hindrance presented by the molecular structure of C_8_H_4_K_2_O_12_Sb_2_ further restricts the direct interaction between Sb^3+^ and the substrate^32^. Nevertheless, the incorporation of MEA can markedly mitigate this limitation. The unique bridging capacity of MEA allows its molecular termini to adhere to the substrate, acting as a molecular bridge that facilitates the connection between Sb^3+^ with the CdS substrate. As shown in Fig. 2c, compared to APT, the [Sb(MEA)]^3+^ complex exhibits a more negative binding energy with the CdS substrate. Hence the bridging effect of MEA significantly enhances the affinity and subsequent binding of Sb^3+^ to the CdS substrate, effectively overcoming the problems posed by the chelating effect of tartrate ions and the steric hindrance of APT. During this process, MEA possesses the capability to sequester free Sb^3+^ within the solution, thereby elevating the likelihood of their interaction with the substrate. This dual functionality not only acts as a physical bridge but also enhances the local concentration of Sb^3+^ in proximity to the substrate surface, increasing the deposition rate of Sb^3+^ on the substrate. Computational analyses have substantiated that [SbMEA]^3+^ can adjust its conformation when it approaches the CdS surface, allowing the O species in MEA to anchor to the Cd site, while the N species connects to the S site on the substrate (Fig. 2d). This structural rearrangement facilitates the binding of [SbMEA]^3+^ to the CdS substrate, thus promoting the nucleation of Sb_2_S_3_ on the substrate. Furthermore, the release of ligands facilitated by the addition of MEA requires minimal energy input, underscoring the high efficiency of MEA in promoting the attachment of Sb^3+^ to the CdS substrate and further the reaction with S^2-^ to form Sb_2_S_3_. Therefore, the MEA additive plays a crucial role in overcoming the challenges faced by APT, culminating in a more efficient and energetically favorable deposition process.

Based on these discussions, the incorporation of MEA into the precursor contributes to the precise control of the deposition conditions, which is essential for the preparation of high-quality Sb_2_S_3_ films. We schematically depict the evolution of the Sb_2_S_3_ deposition process on the substrate with the incorporation of MEA, as shown in Fig. 2e. It is known that, during the initial hydrothermal deposition stage, two typical nucleation processes occur, i.e., homogeneous nucleation in the precursor solution and heterogeneous nucleation on the substrate^19^. In the typical hydrothermal deposition process, it is proposed that the strong chelating effect of APT would result in the formation of stable complexes in the precursor solutions, making it difficult to release Sb^3+^ for effective growth of Sb_2_S_3_ on the substrate^30^. These APT complexes would act as nucleation centers for homogeneous nucleation and growth of Sb_2_S_3_ in the precursor solution. As a result, a large number of suspended Sb_2_S_3_ particles would form in the precursor solution and further deposit onto the substrate during the hydrothermal deposition without the use of additives^27^. This deposition manner is proposed as the particle deposition^30^. In contrast, the incorporation of MEA results in the formation of MEA-complexed Sb^3+^, which would lead to the increase in the concentration of Sb^3+^ and a more uniform distribution in the precursor solution. The deposition process of Sb_2_S_3_ with the presence of MEA complexing agent can be described by Equations S1–S8 (Supplementary Note S1)^33^. According to computational analyses, the unique bridging capacity of MEA leads to a decrease in the energy barrier of heterogeneous nucleation and thus promotes the nucleation of Sb_2_S_3_ on the substrate. This further reduces the sites for homogeneous nucleation in the precursor solution, in good agreement with the reduction of suspended particles in the reactor at the end of hydrothermal deposition (Supplementary Fig. S8). In this case, the incorporation of MEA facilitates the attachment of Sb^3+^ to the substrate and further reaction with S^2-^ to form Sb_2_S_3_, enabling a favorable ion reactive deposition. This is similar to the case where ethylenediaminetetraacetic acid (EDTA) was used as a strong coordination additive to regulate the deposition manner of Sb_2_(S,Se)_3_ films^30^. Moreover, it has been demonstrated that compared to the films grown by the particle deposition mechanism, the films grown via reactive ions are typically associated with larger crystalline size.^34^ Hence, in this work, the addition of MEA into the precursor solution succeeds in controlling the nucleation and growth of Sb_2_S_3_, contributing to the deposition of dense, uniform, large-grained Sb_2_S_3_ films^19^.

Here, to exclude the influence of pH on the deposition rate of Sb_2_S_3_, we measured the pH of the precursor solution without and with the addition of MEA (Supplementary Fig. S9), which reveals that the addition of a small amount of MEA exerts no significant change on the pH of the precursor solution. Therefore, the changes in the morphology and structural properties of Sb_2_S_3_ films should be associated with the addition of MEA. The multifaceted role of MEA plays a pivotal part in regulating the nucleation and growth processes of Sb_2_S_3_ films, resulting in the deposition of ideal absorber films with minimized GB density for constructing solar cells.

Furthermore, we conducted an initial study using DFT calculations to investigate the binding energies and coordination environments of a series of ethanolamine-based additives with varying alkyl chain lengths (Supplementary Figs. S10 and S11). Different alkyl chain lengths can influence the solubility, strength of binding (through inductive effects), steric effects, and the coordination environment around each Sb^3+^ cation, which critically affect film growth. The results demonstrate that, as the alkyl chain length increases, the additives become more favorable for complexing with hydrated Sb^3+^ in the solution (Supplementary Tables S5–S7), and therefore motivates future work to explore these longer-chain ligands, and whether they can also lead to Sb_2_S_3_ films with lower GB density and higher performance.

### Device performance of Sb_2_S_3_ solar cells

**Fig. 3:**
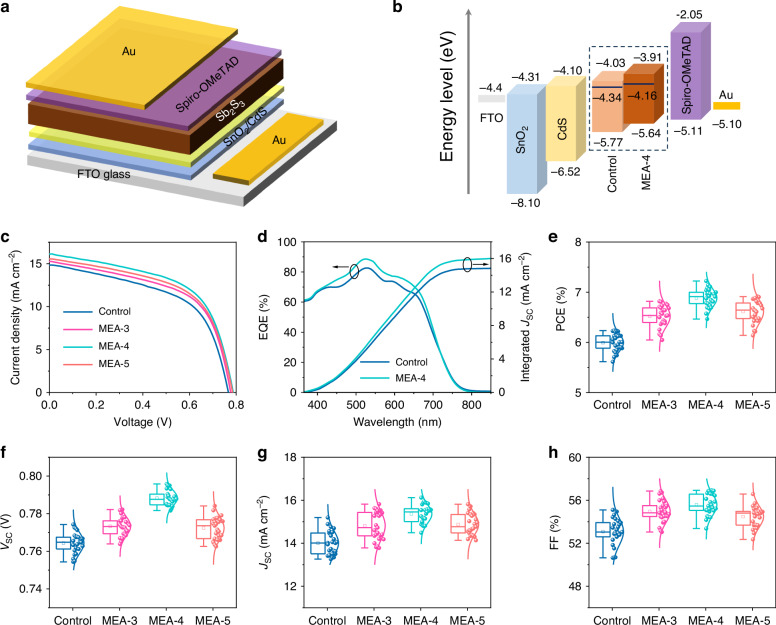
**Photovoltaic performances of Sb**_**2**_**S**_**3**_ **solar cells under AM1.5** **G illumination**. **a** Schematic illustration of the device configuration of planar Sb_2_S_3_ solar cells. **b** Band alignment of the components of Sb_2_S_3_ solar cells. **c** *J–V* curves of best-performing control and MEA-Sb_2_S_3_ solar cells, measured under 1-sun (AM1.5 G, 100 mW cm^−2^) illumination. **d** EQE spectra of the control and MEA-4 Sb_2_S_3_ solar cells. **e**–**h** Statistics of the performance parameters of the control and MEA-Sb_2_S_3_ devices obtained with the addition of different concentrations of MEA

We prepared planar Sb_2_S_3_ solar cells with a typical n-i-p configuration of FTO/SnO_2_/CdS/Sb_2_S_3_/Spiro-OMeTAD/Au, as illustrated in Fig. 3a. The band structure of each functional layer in the Sb_2_S_3_ photovoltaic device is shown in Fig. 3b. The energy levels of Sb_2_S_3_ were obtained from ultraviolet photoelectron spectroscopy (UPS) measurements (Supplementary Fig. S12), while the energy levels of other functional layers are compiled from the literature. The detailed analysis of UPS spectra of the control and MEA-4 Sb_2_S_3_ film samples is given in Supplementary Note S2. The corresponding Fermi level (*E*_F_), valence band maximum and conduction band minimum (CBM) of the control and MEA-4 Sb_2_S_3_ films are summarized in Supplementary Table S8. Here, the bandgap values of the control and MEA-Sb_2_S_3_ films are determined to be 1.74 and 1.73 eV, respectively, based on the Tauc plots of UV-vis-NIR absorption spectra (Supplementary Fig. S13). As shown, the energy levels of Sb_2_S_3_ match well with the electron transport layer (CdS) and HTL (Spiro-OMeTAD), favouring efficient charge transport from absorber layer towards the two electrodes. Moreover, compared to the control Sb_2_S_3_ film, the Fermi level of MEA-Sb_2_S_3_ is shifted up towards the CBM. This indicates an increased electron carrier concentration for n-type Sb_2_S_3_. Compared to the control film, the upward shift of CBM for the MEA-4 film (Fig. 3b) facilitates photogenerated charge-carrier transport from the absorber layer to the electron transport layer. Figure 3c gives current density-voltage (*J–V*) curves of best-performing control and MEA-Sb_2_S_3_ solar cells. As shown, the control device delivers a *V*_OC_ of 766 mV, a *J*_SC_ of 14.89 mA cm^−2^, an FF of 54.49%, and a PCE of 6.22%, while the MEA-4 device yields an enhanced PCE of 7.22%, with a *V*_OC_ of 787 mV, a *J*_SC_ of 16.12 mA cm^−2^, and an FF of 56.92%. That is, the incorporation of MEA affords a 16% relative efficiency enhancement for Sb_2_S_3_ solar cells compared to the control device (or 1% absolute efficiency increase). The performance enhancement of Sb_2_S_3_ solar cells should be associated with the improvements in the materials and optoelectronic properties of the Sb_2_S_3_ absorber films. The corresponding external quantum efficiency (EQE) spectra of both devices are shown in Fig. 3d. It can be seen that both devices exhibit a wide light response ranging from 350 to 750 nm. Compared to the control device, the MEA-4 device exhibits an enhanced EQE value close to 90% at around 520 nm. The obtained integrated *J*_SC_ values are 14.83 and 16.02 mA cm^−2^ for the control and MEA-4 devices, respectively. The values are consistent with the *J*_SC_ obtained from *J–V* curves (within 5% deviation). Figure 3e–h displays the distribution statistics of the PCE, *V*_OC_, *J*_SC_, and FF for the Sb_2_S_3_ devices, and the mean and champion performance parameters are summarized in Table 1. As shown, the photovoltaic performance of Sb_2_S_3_ solar cells first increases and then decreases with an increasing amount of MEA. MEA-Sb_2_S_3_ devices show enhanced photovoltaic performance compared to the control sample, and an optimal device efficiency can be obtained based on the MEA-4 films. The MEA-4 device delivers an enhanced average efficiency of 6.88% in contrast to that of 5.99% for the control device. In addition, the *J–V* curves of as-obtained planar Sb_2_S_3_ solar cells show negligible hysteresis under different scan directions and various scan rates (Supplementary Fig. S14a, b), consistent with those reported in the literature^35,36^.

**Table 1:** Photovoltaic performance parameters of the control and MEA-Sb_2_S_3_ solar cells with the addition of different concentration of MEA, measured under 1-sun (AM1.5 G, 100 mW cm^−2^) illumination

Devices	*V*_OC_ (mV)	*J*_SC_ (mA cm^−2^)	FF (%)	PCE (%)
Control	764 ± 5 (774)	14.01 ± 0.55 (15.19)	53.07 ± 1.27 (55.10)	5.99 ± 0.18 (6.22)
MEA-3	773 ± 4 (782)	14.81 ± 0.66 (15.82)	54.96 ± 1.04 (56.85)	6.52 ± 0.21 (6.81)
MEA-4	788 ± 4 (795)	15.36 ± 0.43 (16.12)	55.57 ± 0.98 (56.92)	6.88 ± 0.18 (7.22)
MEA-5	772 ± 5 (784)	14.87 ± 0.49 (15.81)	54.48 ± 0.96 (56.58)	6.61 ± 0.23 (6.91)

Format: mean ± standard deviation (best value)

It is worth noting that, since the addition of MEA affects the thickness of the Sb_2_S_3_ films, the control and MEA-Sb_2_S_3_ devices were constructed based on absorber films with different thicknesses. In order to reveal the impact of the film thickness on the device performance, we further compared the performance of Sb_2_S_3_ solar cells based on Sb_2_S_3_ films with different thicknesses prepared by using conventional hydrothermal precursor solutions without the addition of MEA. We found that the device based on 430 nm-thick Sb_2_S_3_ films (similar in thickness to the MEA-4 films) has a slight efficiency drop compared to the 339 nm-thick control film (Supplementary Fig. S15a, b). This indicates that the performance enhancement of the MEA-Sb_2_S_3_ devices is not caused by an increase in light absorption. SEM measurements (Supplementary Fig. S15c) further reveal that, compared to the 339 nm-thick control film, the grain size of the 430 nm-thick Sb_2_S_3_ films shows negligible changes. However, the XRD patterns show that the [hk1] preferred orientation of this 430 nm-thick Sb_2_S_3_ film is slightly enhanced compared to the 339 nm-thick film (Supplementary Fig. S15d), similar to the scenario for MEA-Sb_2_S_3_ films. Therefore, it is reasonable to speculate that the enhanced [hk1] orientation of the MEA-4 Sb_2_S_3_ film might be related to the increase in film thickness. The underlying mechanism for the impact of the film thickness on the film orientation needs further study. In addition, a slight drop in the device efficiency was observed when the concentration of MEA increased up to 5 μg mL^−1^, and this might be caused by a continuous increase in the film thickness. To verify this hypothesis, we optimized the thickness of the absorber layer based on the device performance of Sb_2_S_3_ solar cells (Supplementary Fig. S16a, b and Supplementary Table S9). As reflected by the corresponding thickness of the MEA-4 films prepared for different times of hydrothermal growth (Supplementary Fig. S16c–e), the optimal thickness for Sb_2_S_3_ layer is around 428 nm, which can be obtained after 180 min of hydrothermal deposition. Further increases in the film thickness would result in the performance degradation of Sb_2_S_3_ solar cells. Based on such discussions, we conclude that the positive impact of the addition of MEA into the precursor solutions should be responsible for the enhanced performance of MEA-Sb_2_S_3_ solar cells. That is, our proposed explanation is that when the thickness of Sb_2_S_3_ is increased without adding in any MEA, the grain size remains unchanged, and the carrier collection efficiency becomes worse, due to the limited carrier diffusion length. However, when MEA is added, the reduction in GB density results in improved charge-carrier lifetimes (see discussion below on trap density) and mobility (reduced carrier scattering at GBs), such that the carrier collection efficiency is improved for thicker films. But when the film thickness is too high (as in the case for MEA-5), the carrier collection efficiency again reduces, leading to a reduction in *J*_SC_ and FF as the series resistance increases.

Moreover, in “Deposition of high-quality Sb_2_S_3_ films”, we propose that -OH and -NH_2_ groups in MEA contribute to form coordination complexes with Sb^3+^ in the precursor solution. In order to confirm this, we also investigated the use of diethanolamine (DEA) and triethanolamine (TEA), which share similar coordination groups, as the additives in the precursor solution to deposit Sb_2_S_3_ films (Supplementary Fig. S17). The corresponding device performance reveals that the incorporation of DEA and TEA additives also contribute to efficiency improvements of Sb_2_S_3_ solar cells (Supplementary Fig. S18).

**Fig. 4:**
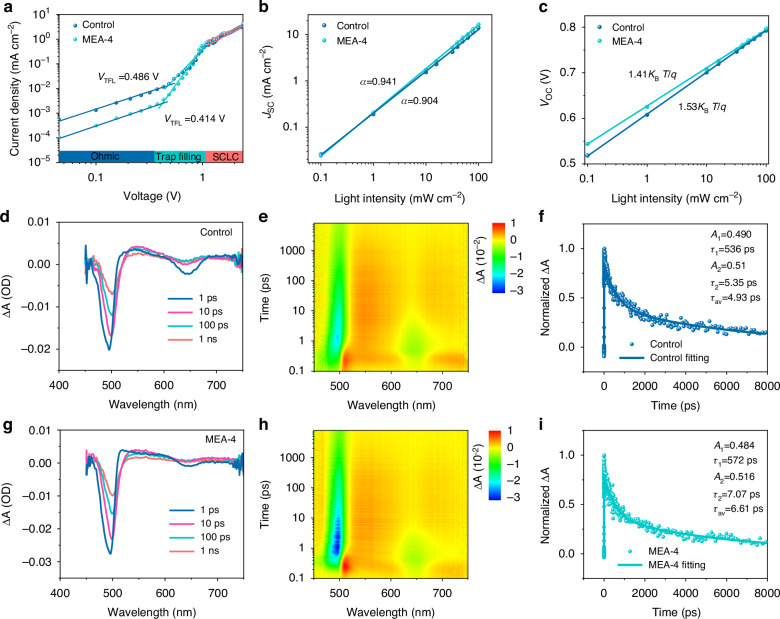
**Device physics and charge-carrier kinetics**. **a** SCLC measurements of the control and MEA-4 devices based on the electron-only device configuration of FTO/CdS/Sb_2_S_3_/PCBM/Au. **b**, **c** The dependence of *J*_SC_ and *V*_OC_ on the light intensity for the control and MEA-4 Sb_2_S_3_ solar cells. **d**, **g** Time-resolved absorption spectra obtained at 1, 10, 100, and 1000 ps, excited by a 400 nm laser pulse, (**e**, **h**) 2D TA spectroscopy pseudo-color images for the time-resolved absorption spectra, and (**f**, **i**) Transient kinetic decay (scatter) and corresponding bi-exponential curve fittings (solid line) monitored at 560 nm of the control and MEA-4 Sb_2_S_3_ films. Δ*A* is defined as the variation of absorption

We further performed in-depth characterization of the Sb_2_S_3_ solar cells to understand the device physics and explore the underlying mechanisms responsible for the performance enhancements. Space charge limited current density measurements of the electron-only devices (FTO/CdS/Sb_2_S_3_/PCBM/Au) are given in Fig. 4a. Generally, the curves can be divided into three parts, the Ohmic region (low voltage), the trap-filled limiting (TFL) region (intermediate voltage), and the trap-free region (high voltage). At the low voltage region, the curve is generally linear. When the voltage exceeds the TFL voltage (*V*_TFL_), the current increases dramatically, implying that the injected carriers have completely occupied the trap states^24^. The *V*_TFL_ values for the control and MEA-4 device are estimated to be 0.486 V and 0.414 V, respectively. The trap state density (*n*_τ_) of Sb_2_S_3_ films can be evaluated according to the equation ${V}_{{\rm{TFL}}}=q{n}_{{\rm{\tau }}}{L}^{2}/2{\varepsilon }_{{\rm{r}}}{\varepsilon }_{0}$, where *q* is the elementary charge, ${n}_{{\rm{\tau }}}$ is the trap state density, *L* is the thickness of the absorber film (here 339 nm and 427 nm for the control and MEA-4 Sb_2_S_3_ films, respectively), ${\varepsilon }_{0}$ is the vacuum permittivity (8.85 × 10^−12 ^F m^−1^), and ${\varepsilon }_{{\rm{r}}}$ represents the relative dielectric constant of Sb_2_S_3_ (6.67). Then the estimated ${n}_{{\rm{\tau }}}$ for the control device and the MEA-4 Sb_2_S_3_ device are 3.12 × 10^15^ and 1.67 × 10^15 ^cm^−3^, respectively. The reduced trap-state density for the MEA-4 sample indicates that the addition of MEA can effectively reduce the density of trap states in Sb_2_S_3_ films^37^. We also measured the dependence of the *J*_SC_ and *V*_OC_ on the light intensity for both devices to reveal the trap-assisted charge recombination loss mechanisms, as shown in Fig. 4b, c. The relationship between *J*_SC_ (or *V*_OC_) and the light intensity *I* can be described by the equations *J*_SC_∝*I*^*α*^ and ${V}_{{\rm{OC}}}\propto (n{k}_{{\rm{B}}}T/q)\mathrm{ln}(I)$, where *I* is the light intensity, *k*_B_ is the Boltzmann constant, *T* is the absolute temperature, *q* is the elementary charge, *α* and *n* reflect the level of charge-carrier recombination^38,39^. The calculated *α* for the control and MEA-4 Sb_2_S_3_ device are 0.904 and 0.941, respectively. Ideally *α* equals 1, and *α* < 1 means the loss of photogenerated carriers caused by the incomplete charge collection. The increment in *α* for the MEA-4 device confirms the improvement of the carrier collection rate^39^. For trap-free solar cells, the slope of *V*_OC_ versus ln(*I*) is *k*_B_*T*/*q*. By performing linear fitting of these curves, the slopes for the control and MEA-4 devices were found to be 1.53*k*_B_*T*/*q* and 1.41*k*_B_*T*/*q*, respectively. The reduced slope for the MEA-4 device indicates that trap-assisted charge-carrier recombination is suppressed. Since the Urbach energy (*E*_U_) is capable of evaluating band tailing, we derived the *E*_U_ values from the EQE spectrum by performing linear fitting of the ln(EQE) vs *E*_U_ plots (Supplementary Fig. S19)^18^. The *E*_U_ values of the control and MEA-4 device were fitted to be 32.36 and 27.98 meV, respectively. The reduction in *E*_U_ suggests the suppression of nonradiative recombination, probably due to a decrease in the defect density, thus explaining the improvement in *V*_OC_ for MEA-Sb_2_S_3_ devices.

The *J–V* curves of the control and MEA-4 solar cells measured under dark conditions are collected to evaluate the carrier recombination and collection in the devices (Supplementary Fig. S20a). According to the abrupt junction *J–V* equation as expressed in Equation (S15), and its formula manipulation as given in Equations (S16–S18), the parameters of diode ideality factor (*A*), the reverse saturation current density (*J*_0_), the series resistance (*R*_S_), and the shunt conductance (*G,* i.e., *R*_SH_^–1^) can be extracted (Supplementary Fig. S20b–d and Supplementary Table S10)^40^. The detailed analysis is given in Supplementary Note S3. It can be seen that, compared to the control device, the MEA-4 device has lower *R*_S_ and *G*, which implies an improvement in the carrier extraction capability. The *A* values extracted from the control and MEA-4 devices are 2.33 and 1.87, respectively; the reduced *A* value indicates the suppression of defect states in the absorber films^23^. The calculated *J*_0_ is reduced from 9.43 × 10^−6 ^mA cm^−2^ for the control device to 1.49 × 10^−6 ^mA cm^−2^ for the MEA-4 device. The *J*_0_ value is related to charge-carrier recombination caused by deep-level defects in the devices, and a reduced *J*_0_ implies the suppression of the defect-induced recombination, contributing to the increase of *V*_OC_ for the MEA-4 device.

Electrochemical impedance spectroscopy was also employed to study the carrier recombination and transport behavior of the devices. The Nyquist plots of the impedance spectra for the control and MEA-4 devices were measured under dark conditions at a bias voltage of 0.7 V (Supplementary Fig. S21), and the resistance and capacitance parameters were obtained by performing curve fitting based on the equivalent circuit diagram given in the inset (Supplementary Table S11). Here *R*_ser_, and *R*_rec_ in the equivalent circuit represent the internal series resistance and recombination resistance, respectively. CPE consists of the interface capacitance (CPE-T) and the ideal capacitance (CPE-P)^41^. *R*_rec_ is related to charge-carrier recombination and its value is equal to the diameter of the semicircle in the Nyquist plots. As shown, the MEA-4 device delivers an increased recombination resistance at the CdS/Sb_2_S_3_ interface (*R*_rec_ = 16.02 kΩ cm^2^) compared to the control device (*R*_rec_ = 9.62 kΩ cm^2^). The significant increase in the recombination resistance is suggested to suppress the charge recombination and improve the charge collection in solar cells.

Ultrafast transient absorption (TA) spectroscopy measurements were performed to understand the charge-carrier kinetics of FTO/SnO_2_/CdS/Sb_2_S_3_ films. As shown in Fig. 4d, g, the TA spectra of both the control sample and the MEA-4 sample display distinct negative ground state bleach (GSB) peaks and positive photoinduced absorption (PIA) peaks. The GSB peaks observed at the wavelengths of 460–510 nm and 610–680 nm can be attributed to the state filling of CdS and the ground state absorption of Sb_2_S_3_, respectively. The PIA peak at the wavelength of 520 to 620 nm can be ascribed to the formation of sulfide radicals (S^−^) as a result of the localization of photogenerated holes on the S atom within the Sb_2_S_3_ lattice^42,43^. The transient dynamics are extracted from the pseudocolor images for the TA spectra of both samples (Fig. 4e, h). The transient kinetic decays monitored at 560 nm for both the control and MEA-4 films are presented in Fig. 4f, i, respectively, which can be well fitted by a phenomenological biexponential equation $y={A}_{1}\exp (-t/{\tau }_{1})+{A}_{2}\exp (-t/{\tau }_{2})$, where *τ*_1_ and *τ*_2_ denote short-lived and long-lived carrier lifetimes. The fitting results are listed in Supplementary Table S12. The gradual decrease of the PIA peak can be attributed to the decay of trapped holes, i.e., the S^−^ species, which we here attribute to the nonradiative carrier recombination in Sb_2_S_3_ films. The MEA-4 sample delivers higher *τ*_av_ values (6.61 ns) compared to the control sample (4.93 ns). The longer lifetime in the MEA-4 sample suggests the suppression of the bulk charge-carrier recombination. Therefore, the TA analysis indicates a slower charge recombination rate in MEA-Sb_2_S_3_ films. The extended lifetime of minority hole carriers would contribute to the enhancement in *V*_OC_^19^.

### IPV performance of Sb_2_S_3_ devices and demonstration for powering IoT electronics

**Fig. 5:**
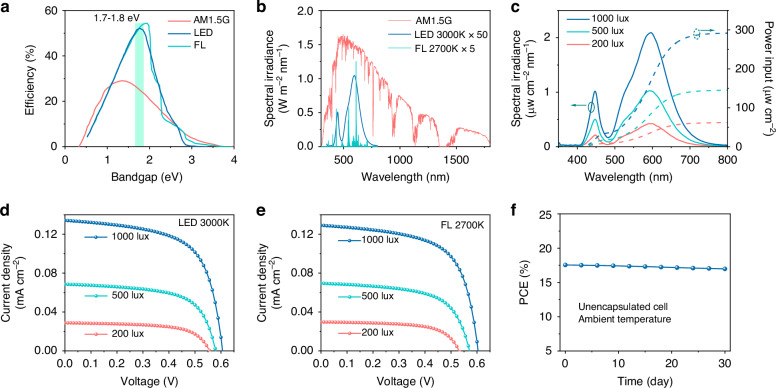
**IPV performance of Sb**_**2**_**S**_**3**_ **solar cells**. **a** Calculated efficiency limit of Sb_2_S_3_ devices under 1-sun (AM1.5 G, 100 mW cm^−2^), 3000 K WLED at 1000 lux, and 2700 K FL at 1000 lux illuminations, respectively^49^. **b** Comparison of the emission spectra of AM1.5 G solar, 3000 K WLED at 1000 lux, and 2700 K FL at 1000 lux. The spectral intensities of WLED and FL are amplified by 50 and 5 times, respectively, in order for these spectra to be legible in this plot. **c** Spectral irradiance and integrated power densities of a 3000 K WLED at 1000, 500, and 200 lux. **d**, **e** *J–V* curves of the MEA-4 Sb_2_S_3_ solar cells measured under the illumination of LED and FL at 1000, 500, and 200 lux. **f** Device stability of unencapsulated MEA-4 Sb_2_S_3_ solar cells stored in the dark under ambient temperature in a cabinet with 15% relative humidity

The bandgaps of the Sb_2_S_3_ films obtained by hydrothermal deposition in this study are 1.73–1.74 eV, which are close to the optimum value of bandgap for IPVs under white light illumination (1.9–2.0 eV)^4^, as shown in Fig. 5a. Figure 5b shows the emission spectra of AM 1.5 G, 3000 K WLED and 2700 K FL, which reveals that the intensity of indoor light sources is 100–1000 times lower than that of 1-sun^5^. Firstly, we measured the emission power spectra of 3000 K WLED and 2700 K FL light sources at 200, 500, and 1000 lux illuminance, as shown in Fig. 5c and Supplementary Fig. S22. The corresponding optical power densities of 3000 K WLED were calculated to be 291.9 μW cm^−2^ (1000 lux), 145.2 μW cm^−2^ (500 lux), 62.0 μW cm^−2^ (200 lux), respectively, while the 2700 K FL delivers 296.7 μW cm^−2^ (1000 lux), 153.1 μW cm^−2^ (500 lux), 64.6 μW cm^−2^ (200 lux), respectively (Supplementary Table S13). All IPV measurements were performed in a black box to prevent the influence of stray light from the environment (Supplementary Fig. S23)^15^. The *J–V* curves for the best-performing MEA-4 Sb_2_S_3_ solar cell measured under WLED and FL illumination are shown in Fig. 5d, e, respectively. The corresponding IPV performance parameters are summarized in Table 2. As shown, under the WLED illuminations of 1000, 500, and 200 lux, the PCEs of this device are 17.55%, 17.17%, and 16.41%, respectively, with the output power densities being 51.23, 24.93, 10.18 μW cm^−2^, respectively; under the FL illuminations of 1000, 500, and 200 lux, the device delivers the PCEs of 16.54%, 15.92%, and 15.51%, respectively, with the output power densities being 49.09, 24.37, 10.02 μW cm^−2^, respectively. As expected, Sb_2_S_3_ solar cells exhibit excellent IPV performance under low-intensity indoor light illuminations. To the best of our knowledge, the IPV efficiency of 17.55% is the highest reported thus far for Sb_2_S_3_ photovoltaics under indoor lighting. Compared to a broad class of emerging perovskite-inspired materials for IPVs, including Ag-Bi-I compounds, BiOI and vacancy-ordered triple perovskites, etc. (Supplementary Table S14), this work demonstrating >17% PCE for Sb_2_S_3_ solar cells is an important advance in the field. The dependences of photovoltaic parameters (PCE, *V*_OC_, *J*_SC_, FF, *R*_S_, and *R*_SH_) of the MEA-4 device on the indoor light intensity are depicted in Supplementary Fig. S24. As shown, *J*_SC_ is linearly proportional to the illumination intensity, *V*_OC_ decreases approximately logarithmically with the illumination intensity, and FF involves very small changes under such low illumination intensities. This enables the Sb_2_S_3_ device to yield the best IPV efficiency under 1000 lux. Moreover, compared to the device performance measured under the standard AM1.5 G illumination, the low-intensity illumination leads to a significant reduction in *J*_SC_ and *V*_OC_ and a remarkable enhancement in FF under the indoor light illumination^44^. Such photovoltaic parameters follow similar trends as those previously reported in the literature^16^. The detailed discussions are given in Supplementary Note S4. We further recorded the device stability of the unencapsulated Sb_2_S_3_ solar cell stored in a cabinet with 15% relative humidity at ambient temperature (Fig. 5f). The device retained 96.75% of its initial efficiency after one-month storage. The excellent device stability under indoor environments demonstrates the great prospect of Sb_2_S_3_ solar cells for IoT applications.

**Table 2:** Indoor photovoltaic performance of MEA-4 Sb_2_S_3_ solar cells measured under WLED (3000 K) and white light FL (2700 K) at 1000, 500, and 200 lux

MEA-4 device	*V*_OC_ (mV)	*J*_SC_ (mA cm^−2^)	FF (%)	PCE (%)	*R*_S_ (kΩ cm^2^)	*R*_SH_ (kΩ cm^2^)
1-sun	787	16.12	56.92	7.22	0.007	0.23
1000 lux LED	606	0.134	63.09	17.55	0.44	48.31
500 lux LED	579	0.0685	62.86	17.17	0.76	86.57
200 lux LED	557	0.0289	63.22	16.41	2.17	206.66
1000 lux FL	604	0.129	63.00	16.54	0.44	49.47
500 lux FL	571	0.0696	61.32	15.92	0.90	75.55
200 lux FL	531	0.0297	63.54	15.51	1.97	226.47

**Fig. 6:**
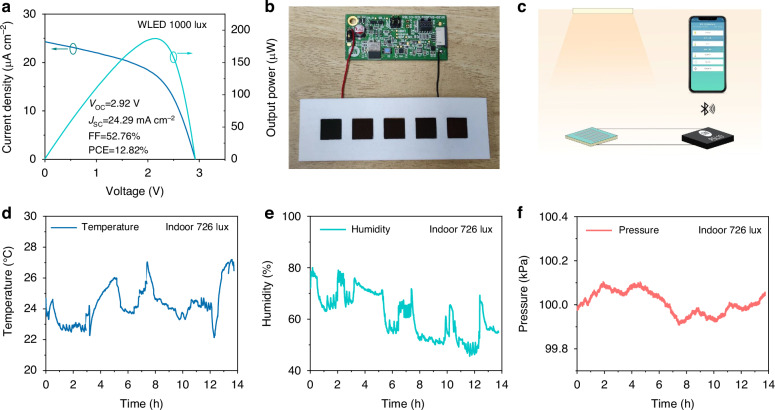
**Demonstration for powering IoT electronics**. **a** *J–V* curve of Sb_2_S_3_ solar minimodule (5 × 1 cm^2^). **b** The picture showing the connection between the sensor platform and the Sb_2_S_3_ minimodule. **c** Schematic circuit diagram of an IPV-driven sensor device. **d**–**f** Temperature, humidity, and atmospheric pressure variations recorded by the IPV-driven sensor in the office with the 726 lux indoor illumination for nearly 14 h

Having developed efficient Sb_2_S_3_ IPVs, we are now in the position to prototype these for the first time for powering IoT wireless electronics. It is known that a large number of IoT devices typically require very little energy with power consumption ranging from several μW to a few mW, which depends on the application and communication protocol^6^. In this work, we used the RSL 10 solar cell multi-sensor platform (RSL10-SOLARSENS-GEVK) manufactured by ON Semiconductor, which is a comprehensive development platform for battery-less IoT applications in smart buildings, smart homes, and industrial sectors (Supplementary Fig. S25). This board is based on the industry’s lowest-power Bluetooth® low-power radio (RSL10) and features sensors for environmental sensing: the BME280 smart environmental sensor. The platform also features a lightweight, low-profile 47 F storage capacitor, a programming and debugging interface, and an interface to a solar cell. To drive this sensor platform via an external power supply, a solar module with a *V*_OC_ higher than 2.65 V and an average input power exceeding 50 μW is needed. The *J–V* curve of as-prepared 1 cm^2^ area MEA-4 Sb_2_S_3_ solar cell (Supplementary Fig. S26) demonstrates a PCE of 15.77% under 1000 lux WLED illumination, coupled with a *V*_OC_ of 0.596 V, a *J*_SC_ of 124 μA cm^−2^ and an FF of 62.39%. Compared with the 0.06 cm^2^ small-area cell, the device performance of this 1 cm^2^ area device only shows about a 10% decrease in PCE. We then obtained an Sb_2_S_3_ minimodule (5 × 1 cm^2^) by connecting five Sb_2_S_3_ devices together in series. As shown in Fig. 6a, the *J–V* curve of this Sb_2_S_3_ minimodule achieves a PCE of 12.82% with a *V*_OC_ of 2.92 V under 1000 lux WLED illumination. This minimodule produces an output power of 187.17 μW at the maximum power point (*V*_max_ = 2.17 V). Compared with commercial a-Si:H IPV products in the market, the Sb_2_S_3_ minimodule here is of competitive performance in terms of PCE, *V*_OC_ and output power^2,11^. The connection between the sensor and the Sb_2_S_3_ minimodule is shown in Fig. 6b. Figure 6c illustrates an IPV-driven sensor device, in which the Sb_2_S_3_ minimodule converts light energy into electrical energy and drives the sensor to operate stably. The sensor communicates with the mobile phone via Bluetooth Low Energy, which enables real-time monitoring and collection of environmental data. Supplementary Fig. S27 presents plots of the temperature, humidity, and atmospheric pressure measured in the laboratory by the Sb_2_S_3_ minimodule-driven sensor platform running continuously under 1000 lux WLED illumination for 140 min in ambient air without encapsulation. Furthermore, we placed it on a desk in a common office area (with a light intensity of ~726 lux) to successfully monitor the environmental parameters for nearly 14 h, as shown in Fig. 6d–f, confirming that the Sb_2_S_3_ minimodule can drive IoT devices under indoor light continuously and stably.

## Discussion

In this work, we demonstrate an effective additive engineering strategy to improve the optoelectronic properties of Sb_2_S_3_ films and enhance the photovoltaic performance of corresponding planar Sb_2_S_3_ solar cells. The multifaceted role of MEA plays a pivotal part in regulating the nucleation and growth of Sb_2_S_3_ films for enhanced heterogeneous nucleation on the substrate. Compared to the control sample, the MEA-modulated Sb_2_S_3_ films show reduced GB density, optimized band positions, and increased carrier concentration, affording suppressed charge-carrier recombination and efficient charge collection in solar devices. The corresponding MEA-Sb_2_S_3_ solar cell achieves a state-of-the-art efficiency of 7.22% under AM1.5 G illumination, much higher than that of the control device (6.22%). This device delivers an IPV efficiency of 17.55% under 1000 lux WLED illumination, which, to our knowledge, is the highest reported so far for Sb_2_S_3_ solar cells. Furthermore, we constructed an Sb_2_S_3_ minimodule (5 × 1 cm^2^) and successfully reported the use of this module to power IoT wireless devices for the first time. This study demonstrates the broad prospect of Sb_2_S_3_ solar cells for IPV applications.

## Materials and methods

### Materials

Ethanol (99.0%), acetone (99.0%), methanol (99.0%), ammonia solution (25%–28%, in water), thiourea (99.0%), Cd(NO_3_)_2_·4H_2_O (99.0%), CdCl_2_·2.5H_2_O (99.0%), C_4_H_4_KO_7_Sb·0.5H_2_O (99.0%), and Na_2_S_2_O_3_·5H_2_O (99.0%) were purchased from Sinopharm. SnO_2_ colloidal dispersion (15.0% in H_2_O) was purchased from Alfa Aesar. MEA (99.0%), acetonitrile (99.9%), chlorobenzene (99.5%), and tert-butylpyridine (TBP, 96%) were purchased from Aladdin. Spiro-OMeTAD (99.8%) and lithium bis(trifluoromethylsyfonyl)imide salt (99.95%) were purchased from Xi’an Polymer Light Technology. All the chemicals and materials were purchased and used as received.

### Device fabrication

Firstly, the SnO_2_/CdS electron transport layer was deposited onto Fluorine-doped Tin Oxide (FTO, ~15 Ω sq^−1^) conductive substrates. The corresponding detailed fabrication processes were referred from our previous work^19^. Secondly, the Sb_2_S_3_ thin films were deposited onto FTO/SnO_2_/CdS substrates via a hydrothermal method. For a typical hydrothermal precursor solution, C_4_H_4_KO_7_Sb·0.5H_2_O and Na_2_S_2_O_3_·5H_2_O were employed as antimony and sulfur sources, respectively. A 40 mL mixture solution of 20 mM C_4_H_4_KO_7_Sb·0.5H_2_O and 80 mM Na_2_S_2_O_3_·5H_2_O were prepared in deionized (DI) water, and further added into a 50 mL Teflon tank. For the case with the addition of MEA, a certain amount of MEA additive (3 μg/mL, 4 μg/mL, and 5 μg/mL) was added into the above mixture solution. The FTO/SnO_2_/CdS substrates were immersed into the precursor solution with the conducting side facing down inside the tank and tilted at an angle of ~75° to the wall of the Teflon tank. The tank was then sealed and kept at 135 °C for 180 min. When the reaction ended and the tank cooled down, the substrates were taken out, followed by rinsing with DI water and drying with N_2_ flow. Then, as-deposited films were annealed at 370 °C for 10 min in a N_2_ filled glovebox. Thirdly, the Spiro-OMeTAD layer was spin-coated onto the Sb_2_S_3_ layer as the HTL, and an Au layer of ~60 nm thickness was further evaporated as the back electrode. The detailed deposition process can be found in the literature^19,24^. The active area of devices was 0.06 cm^2^ as defined by a mask.

### Characterization

The crystal structure of film samples were revealed by using XRD characterization performed on a diffractometer (X’Pert PRO MPD, *λ* = 1.54056 Å). SEM images were collected on a JEOL field emission scanning electron microscope (JSM-6700F), and AFM images were performed on a Bruker atomic force microscope (Dimension Icon). The Raman spectra spectroscopy was performed on measured on a LabRam HR Evolution (HORIBA JOBIN YVON). Absorption spectra were collected using a CARY 5000 Agilent spectrophotometer. XPS characterzation was performed using a Thermo Scientific K-Alpha spectrometer. UPS measurements were conducted on a Thermo ESCALAB XI instrument. The *J–V* curves of solar cells were measured under 1-sun illumination (AM 1.5, 100 mW cm^−2^) using a Newport Oriel Sol 3 A Solar Simulator, combined with a Keithley 2400 digital source meter. The *J–V* curves were performed in the reverse direction (open-circuit to short-circuit) with a scanning rate of 370 mV s^−1^ (voltage step of 11.1 mV and delay time of 30 ms) unless otherwise stated. The EQE spectra were performed by using an electrochemical workstation (Zennium Pro., Zahner) equipped with a CIMPS-IPCE system and Thales software, measured under the direct current (DC) mode, and a tunable LED lamp (365–1020 nm, TLS03) was used as the light source. The impedance spectra were acquired on a Zahner workstation (Zennium Pro.) under an applied voltage of 0.7 V, a scanning range of 0.5 Hz to 1 MHz, and an AC amplitude of 20 mV under dark, and the data were then fitted using Z-view software. The ultrafast TA properties of films were investigated based on a Helios Ultrafast pump-probe system. For this system, a nondegenerate pump-probe configuration was employed to probe the transient dynamics within the femtosecond to nanosecond time range (50 fs to 7 ns) under ambient conditions. The pump pulses at a wavelength of 400 nm were generated by doubling the 800 nm pulse using a beta barium borate (BBO) crystal on an optical parametric amplifier. The white light continuum probe pulses were formed by 800 nm femtosecond with a 2 mm sapphire plate for the 400–800 nm range. The IPV efficiencies of Sb_2_S_3_ solar cells and minimodules were measured on a home-made platform (Supplementary Fig. S23). The lux levels of LEDs (Ccobalance T5/5w) and FLs (NVC YZ08-T5/8w) were obtained by using a luxmeter (TES-1330A), and the spectral irradiance of the indoor light source of 3000 K LED and 2700 K FL were measured with a high-precision fiber-optic spectrometer (Maya2000 Pro, Ocean Optics). The corresponding indoor light power densities were calculated by integrating the spectral irradiance under different illumination intensities (i.e., 200, 500, 1000 lux) over the entire wavelength range. The spectrometer was calibrated by B. Hays in compliance with the U.S. National Institute of Standards and Technology practices recommended in the NIST Handbook 150-2E, Technical Guide for Optical Radiation Measurements. The optical resolution of the spectrometer was about 1.1 nm, and the spectral wavelength accuracy was ±1.4 nm.

### Calculations

The geometric optimization and energy calculations were carried out by DFT calculations. The formation energy of [Sb(MEA)_3_]^3+^ was performed using DMol^3^ package^45^, employing the Perdew-Bruke-Ernzerhof (PBE) functional of the general gradient approximation (GGA)^46^, including p polarization (DNP) and all-electron relativistic effects. Convergence criteria of energy, maximum force and maximum displacement were set to 1 × 10^−5^ hartree, 0.002 hartree Å^−1^ and 0.005 Å, respectively. Additionally, orbital occupation smearing with a value of 0.005 hartree was applied to expedite convergence. The binding energy calculations of the Sb^3+^ complexes on the CdS substrate were conducted using the Vienna ab initio simulation package^47^. A plane wave basis set with an energy cutoff of 450 eV, projector augmented wave pseudopotentials^48^, and the generalized gradient approximation for exchange-correlation functional were employed^46^. The Brillouin zone of the supercell model was sampled by a 2 × 2 × 1 uniform k-point mesh. The CdS (100) surface was simulated using a supercell consisting of a three-layer slab^19^. A vacuum layer of 15 Å along the *z*-direction was implemented to prevent slab interactions. Furthermore, the binding energy and ligand dissociation energy of Sb^3+^ from [SbMEA]^3+^ were calculated during the deposition process of Sb_2_S_3_. The calculating formula and detailed details for the formation energy and the binding energy can be found in Supplementary Note S5 and Supplementary Table S1–7.

## Supplementary information

Supplementary Information

## Data Availability

The data that support the findings of this study are available from the Oxford University Research Archive, with the 10.5287/ora-z52dx6xxq.
