# Supplementary material for: Additive engineering for Sb2S3 indoor photovoltaics with efficiency exceeding 17%
Source: Light Sci Appl. 2024 Oct 2;13:281. doi: 10.1038/s41377-024-01620-0 (PMC11447099; doi:10.1038/s41377-024-01620-0)
Supplement: Supplementary file 1 — Supplementary Information [file 41377_2024_1620_MOESM1_ESM.pdf]

Supplementary Information for

Additive engineering for Sb_2S_3 indoor photovoltaics with efficiency exceeding 17%

Xiao Chen,[1] Xiaoxuan Shu,[2] Jiacheng Zhou,[1] Lei Wan,[1] Peng Xiao,[3] Yuchen Fu,[4] Junzhi Ye,[4] Yi-Teng Huang,[4] Bin Yan,[5] Dingjiang Xue,[5] Tao Chen,[3] Jiejie Chen*,[2] Robert L. Z. Hoye,*,[4] and Ru Zhou*,[1,4]

[1] School of Electrical Engineering and Automation, Hefei University of Technology, Hefei 230009, P. R. China

[2] Key Laboratory of Urban Pollutant Conversion, Department of Environmental Science and Engineering, University of Science & Technology of China, Hefei 230009, P. R. China

[3] Hefei National Research Center for Physical Sciences at the Microscale, School of Chemistry and Materials Science, University of Science and Technology of China, Hefei 230026, P. R. China

[4] Inorganic Chemistry Laboratory, Department of Chemistry, University of Oxford, South Parks Road, Oxford OX1 3QR, United Kingdom

[5] Beijing National Laboratory for Molecular Sciences, CAS Key Laboratory of Molecular Nanostructure and Nanotechnology, Institute of Chemistry, Chinese Academy of Sciences, Beijing 100190, P. R. China

---

* Corresponding authors:

chenjiej@ustc.edu.cn (Jiejie Chen)

robert.hoye@chem.ox.ac.uk (Robert L. Z. Hoye)

zhouru@hfut.edu.cn (Ru Zhou)

Supplementary Note 1:

The deposition of Sb_2S_3 with the presence of MEA as complexing agent can be described by Equations (S1-S8)[1,2]:

(1) The release of S^{2-}:

$$Na_2S_2O_3 \rightarrow 2Na^+ + S_2O_3^{2-} \quad (S1)$$

$$2S_2O_3^{2-} \rightarrow S_4O_6^{2-} + 2e^- \quad (S2)$$

$$S_2O_3^{2-} + H^+ \rightleftharpoons S + HSO_3^- \quad (S3)$$

$$S + 2e^- \rightarrow S^{2-} \quad (S4)$$

(2) The release of Sb^{3+}:

$$\left[Sb_2(C_4H_2O_6)_2\right]K_2 \rightarrow \left[Sb_2(C_4H_2O_6)_2\right]^{2-} + 2K^+ \quad (S5)$$

$$\left[Sb_2(C_4H_2O_6)_2\right]^{2-} + 4H^+ \rightarrow 2Sb^{3+} + 2(C_4H_4O_6)^{2-} \quad (S6)$$

(3) The formation of complexing agent:

$$Sb^{3+} + 3MEA \rightleftharpoons [Sb(MEA)_3]^{3+} \quad (S7)$$

(4) The formation of Sb_2S_3:

$$2Sb^{3+} + 3S^{2-} \rightarrow 2Sb_2S_3 \quad (S8)$$

Supplementary Note 2:

The VBM and CBM of Sb_2S_3 and MEA-4 Sb_2S_3 films can be derived from the intersection of the tangent line and the coordinate axis at the secondary electron cut-off and near the Fermi edge, as shown in Figure S12. The cut-off binding energy (E_{cutoff}) values of Sb_2S_3 and MEA-4 Sb_2S_3 are 16.88 and 17.05 eV, respectively. According to Equation (S9), the work function (*WF*) can be determined by:

$$WF = E_{cutoff} - 21.22 \quad (S9)$$

The *WF* values relative to the Fermi level of Sb_2S_3 and MEA-4 Sb_2S_3 are calculated to be -4.34 eV and -4.16 eV, respectively. The VBM relative to the Fermi level of control Sb_2S_3 and MEA-4 Sb_2S_3 are -1.43 eV and -1.48 eV, respectively. Therefore, the VB

positions (E_{VB}) relative to the vacuum level of control Sb_2S_3 and MEA-4 Sb_2S_3 are calculated to be -5.77 and -5.64 eV, respectively, according to Equation (S10):

$$E_{VB} = WF - E_{F,edge} \tag{S10}$$

Combined with the band gap value, the CB positions relative to the vacuum level of the control Sb_2S_3 and MEA-4 Sb_2S_3 are -4.03 eV and -3.91 eV, respectively.

Supplementary Note 3:

According to the abrupt junction *J-V* equation as expressed in Equation (S11), and its formula manipulation as given in Equations (S12-S14), the parameters of junction ideality factor (*A*), the reverse saturation current density (J_0), the series resistance (R_s), and the shunt conductance (*G,* i.e., $1/R_{sh}$)) can be extracted[3]:

$$J = J_0 \exp[\frac{q}{AKT}(V - JR_s)] + GV - J_L \tag{S11}$$

$$\frac{dJ}{dV} = \frac{q}{AKT} \times J_0 \exp[\frac{q}{AKT}(V - JR_s)] + G \tag{S12}$$

$$\frac{dV}{dJ} = R_s + \frac{AKT}{q} \times \frac{1 - G\frac{dV}{dJ}}{J + J_L - GV} \tag{S13}$$

$$\ln(J + J_L - GV) = \frac{q}{AKT} \times (V - R_s J) + \ln J_0 \tag{S14}$$

Supplementary Note 4:

Under the low-intensity indoor illuminations, J_{SC} involves a typical linear decline with the decrease of the illumination intensity, following the relation $J_{SC} = J_{SC,1000lux} \times [I_{illum}/I_{illum,1000lux}]$, where $J_{SC,1000lux}$ is the short-circuit current density under 1000 lux, I_{illum} is the indoor illumination intensity, and $I_{illum,1000lux}$ is the light intensity under 1000 lux. V_{OC} decreases approximately logarithmically with the illumination intensity, in good agreement with the equation $V_{OC} \propto nk_B T / q \times [I_{illum}/I_0]$, where *n* is the ideality factor of the junction diode, *q* is the electron charge, k_B is the Boltzmann's constant, *T* is the absolute temperature, and I_0 is the reverse saturation

current. Such discussions also explain the decrease in J_{SC} and V_{OC} under low-intensity illuminations compared to that measured under the standard AM1.5G (100 mW cm^{-2}) illumination. Different from the downward trend for J_{SC} and V_{OC} with the decrease of the illumination intensity, FF involves a remarkable enhancement under the indoor light illumination in contrast to the standard AM 1.5G illumination[4]. This scenario can be explained as follows. According to the literature, FF depends on the series resistance R_S and shunt resistance R_{SH}, which represent the parameters of the equivalent circuit model for a solar cell. The R_S and R_{SH} can be calculated from the line slope of the *J*-*V* curves at the open-circuit voltage and short-circuit current points, respectively. The impacts of R_S and R_{SH} on FF can be separately expressed by the following empirical expressions[5,6]:

$$\mathrm{FF}_0 = \frac{v_{\mathrm{OC}} - \ln(v_{\mathrm{OC}} + 0.72)}{v_{\mathrm{OC}} + 1} \tag{S15}$$

$$\mathrm{FF}_\mathrm{S} = \mathrm{FF}_0 (1 - r_\mathrm{S}) \tag{S16}$$

$$\mathrm{FF}_\mathrm{SH} = \mathrm{FF}_0 \left[1 - \frac{(v_{\mathrm{OC}} + 0.7)}{v_{\mathrm{OC}}} \frac{\mathrm{FF}_0}{r_{\mathrm{SH}}} \right] \tag{S17}$$

$$\mathrm{FF} = \mathrm{FF}_0 (1 - r_\mathrm{S}) \left[1 - \frac{(v_{\mathrm{OC}} + 0.7)}{v_{\mathrm{OC}}} \frac{\mathrm{FF}_0 (1 - r_\mathrm{S})}{r_{\mathrm{SH}}} \right] \tag{S18}$$

where v_{OC} is the normalized V_{OC} ($v_{OC} = qV_{OC} / nk_B T$, where *n* is the diode ideality factor, k_B is the Boltzmann's constant, and *T* is the absolute temperature), r_S is the normalized series resistance ($r_S = R_S / R_{ch}$ with $R_{ch} = V_{OC} /(J_{SC} \cdot A)$, where R_{ch} is characteristic resistance for the device, and *A* is the effective area of the device), and r_{SH} is the normalized shunt resistance ($r_{SH} = R_{SH} / R_{ch}$). As shown, the variation of FF with the illumination intensity results from the combined effect from R_S and R_{SH}. Moreover, it has been demonstrated that the FF at low irradiance will be dominated by the R_{SH} since $r_S \approx 0$. Hence, compared to the case of AM1.5G illumination, the

increase of R_{SH} contributes to the increase of FF measured under low-intensity indoor illumination. This might be associated with the fact that, under indoor illumination, the photogenerated carrier density is much lower than that under AM 1.5G, so the charge-carrier recombination is reduced, which might be the reason for the higher R_{SH} and FF under the indoor illumination[5,7].

Supplementary Note 5:

We proposed that both O and N atoms of MEA could potentially coordinate with Sb^{3+} based on their availability and affinity in the complex formation process. To address the differences in formation energies for these coordination scenarios (Figure s6 and Table S1), we compared the formation energies of Equation (S19) for three different coordination scenarios and predicted the most favorable coordination configurations by DFT calculations.

$$[Sb(H_2O)_3]^{3+} + MEA \rightarrow [SbMEA]^{3+} + 3H_2O \quad (S19)$$

The results indicate that there are indeed differences in formation energies when MEA coordinates through O versus N atoms. Specifically, the coordination via O atoms typically shows a lower formation energy compared to N atoms, suggesting a more stable complex formation. This aligns with the higher electronegativity and stronger binding affinity of oxygen compared to nitrogen.

We also hypothesize that MEA could potentially coordinate with Sb^{3+} through both O and N atoms simultaneously, forming a more stable complex with potentially lower overall formation energy. The results reveal that simultaneous coordination of O and N atoms of MEA to Sb^{3+} results in the lowest formation energy compared to single-atom coordination scenarios. This dual coordination could enhance the stability of the complex.

The formation energy of $[Sb(MEA)_3]^{3+}$ is expressed as:

$$E_{\mathrm{formation\_[Sb(MEA)_n]^{3+}}} = E_{\mathrm{[Sb(MEA)_n]^{3+}}} - E_{\mathrm{[Sb(H_2O)_3]^{3+}}} - nE_{\mathrm{MEA}} + 3E_{\mathrm{H_2O}} \quad (S20)$$

where $E_{\mathrm{[Sb(MEA)_n]^{3+}}}$ represents the energy of compound, $E_{\mathrm{[Sb(H_2O)_3]^{3+}}}$, E_{MEA} and

E_{H_2O} denote the energies of the simple substances of $[Sb(H_2O)_3]^{3+}$, MEA and H_2O, respectively, composing $[Sb(MEA)_n]^{3+}$. Here, n represents the number of different elements in a unit compound.

The binding energy is determined as:

$$E_{\text{binding}} = E_{*\text{A}} - E_{*} - E_{\text{A}} \tag{S21}$$

where $E_{*\text{A}}$, E_{*} and E_{A} represent the energies of the surface-adsorbate, free adsorbate, and bare substrate, respectively.

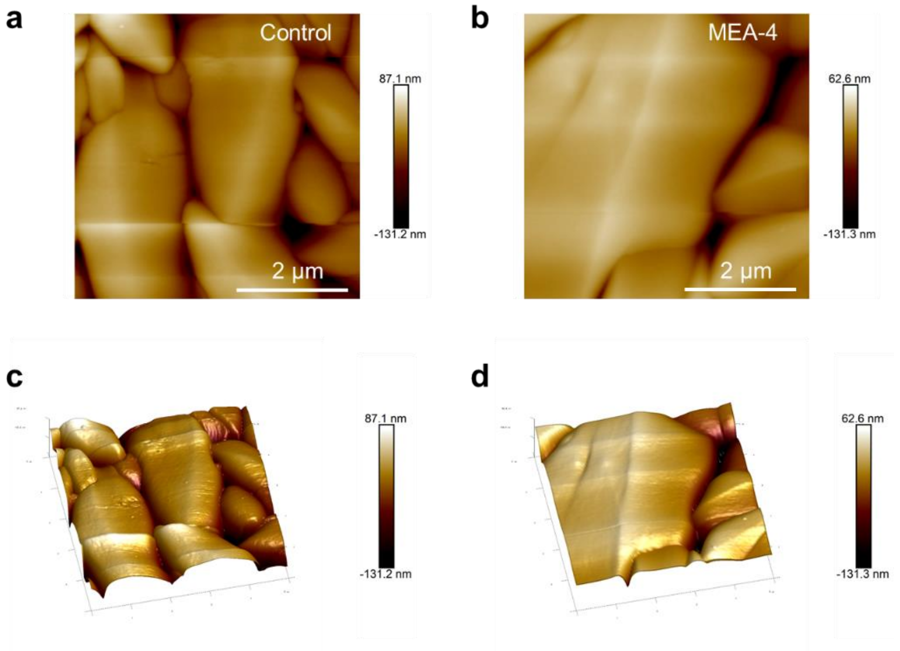

Figure S1. **a**, **b** 2D and **c**, **d** 3D topography spatial maps of the control film sample and MEA-4 Sb_2S_3 film sample.

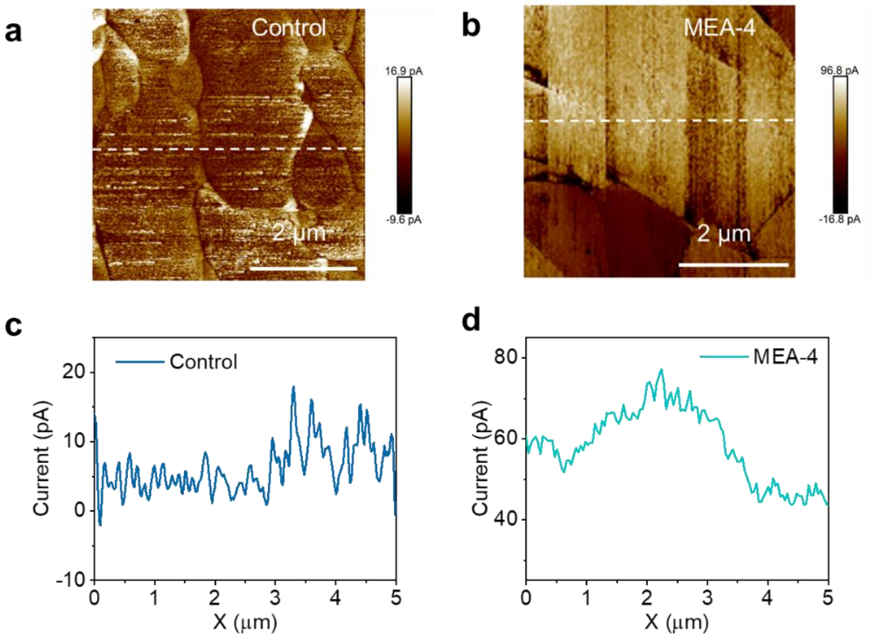

Figure S2. **a**, **b** c-AFM images and **c**, **d** corresponding height profile and current analysis of the control film sample and MEA-4 Sb_2S_3 film sample.

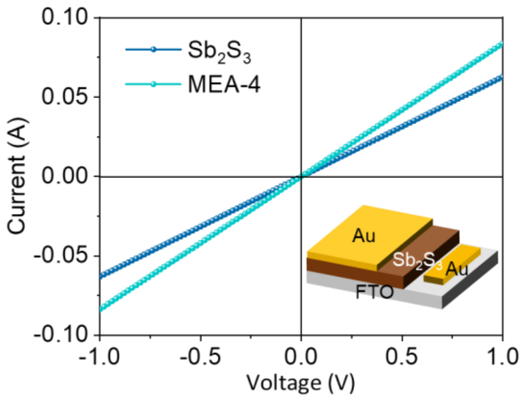

Figure S3. The *I-V* curve measurements of the devices with the configuration of FTO/Sb_2S_3/Au to estimate the electrical conductivity of the control film sample and MEA-4 Sb_2S_3 film sample.

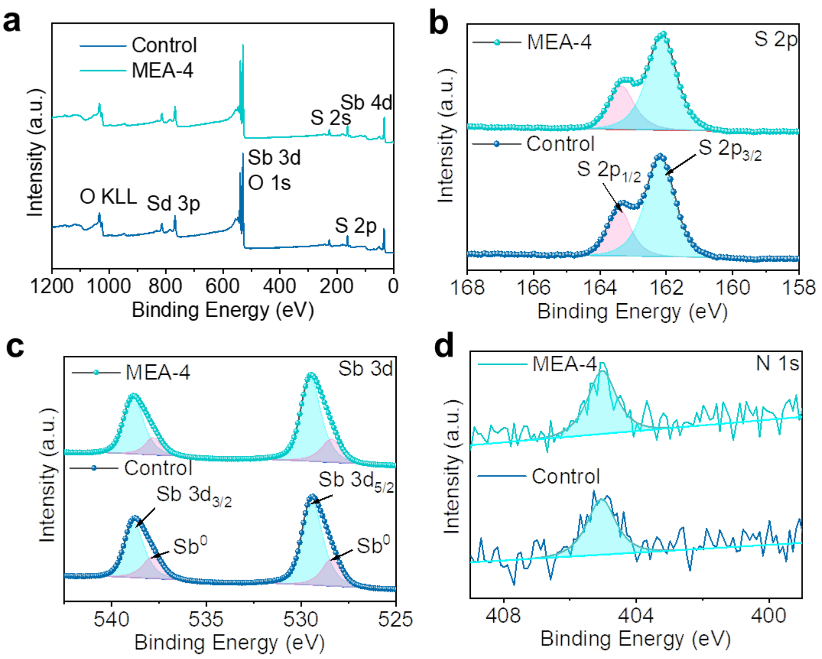

Figure S4. a The survey XPS spectra for the control and MEA-4 film samples, and high-resolution XPS spectra and deconvolution analysis of **b** S 2p, **c** Sb 3d and **d** N 1s for the control and MEA-4 film samples prepared by 180 min-hydrothermal deposition. The surfaces of both films were etched by Ar^+ sputtering to eliminate the oxidation layer. As shown, the Sb spectra show two main peaks at 538.9 eV and 529.5 eV, which are assigned to Sb $3d_{3/2}$ and Sb $3d_{5/2}$, respectively.[8] Two minor peaks located at 537.8 eV and 528.5 eV correspond to the presence of Sb metal (Sb^0) caused by Ar^+ sputtering.[9] The characteristic peaks at 163.4 eV and 162.2 eV can be indexed to S $2p_{1/2}$ and S $2p_{2/3}$, respectively.[10] The N spectra show a characteristic peak at 405.1 eV, which corresponds to the adsorption of N_2.[11,12] Overall, both films share nearly identical XPS peaks without obvious peak shifts, indicating that the addition of MEA has no impacts on the chemical states of Sb_2S_3.

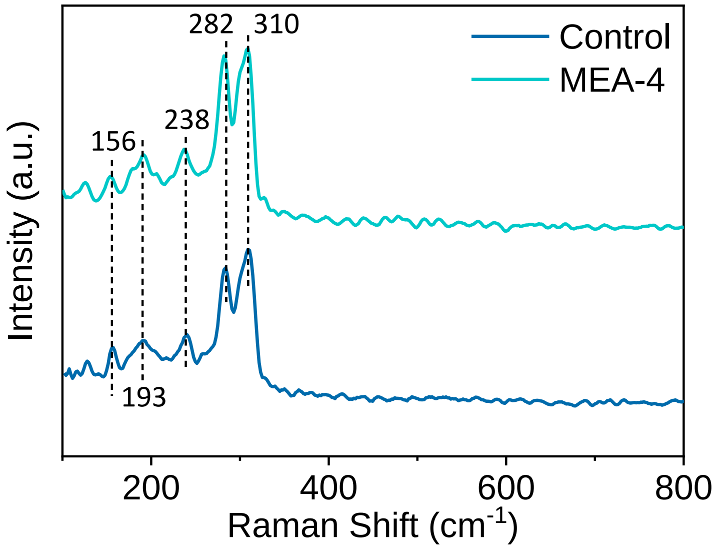

Figure S5. Raman spectra of the control films and MEA-4 Sb_2S_3 films. The 156, 193, and 282 cm^{-1} peaks are correlated with the A_g modes of the pyroxene Sb_2S_3, while the 238 and 310 cm^{-1} peaks are assigned to the B_{1g} vibrational modes.[13] The Raman spectra reveal the identical characteristic peaks for the control and MEA-4 samples.

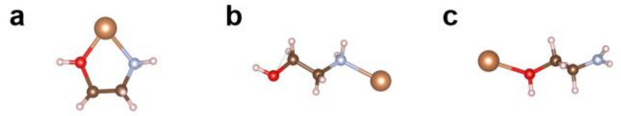

Figure S6. The structures of $[SbMEA]^{3+}$ for three different coordination scenarios. (a) N atom and O atom, (b) Only N atom, and (c) Only O atom

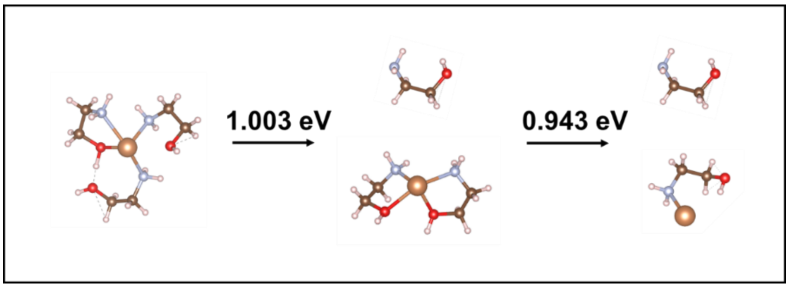

Figure S7. The ligand dissociation energies of $[SbMEA]^{3+}$ from $[Sb(MEA)_3]^{3+}$

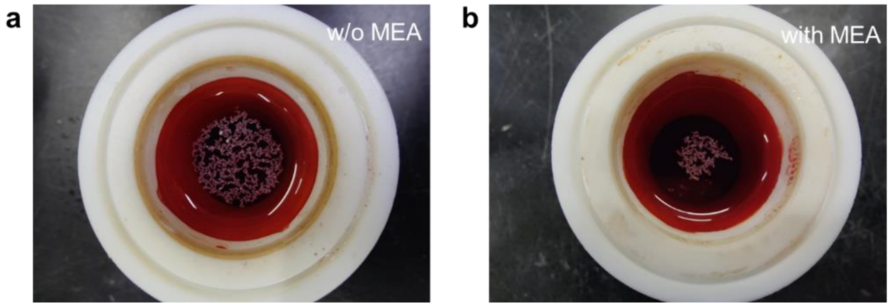

Figure S8. Digital images of hydrothermal deposition phenomena for the cases without MEA (left) and with MEA (right) in the precursor solutions, collected just after the hydrothermal process.

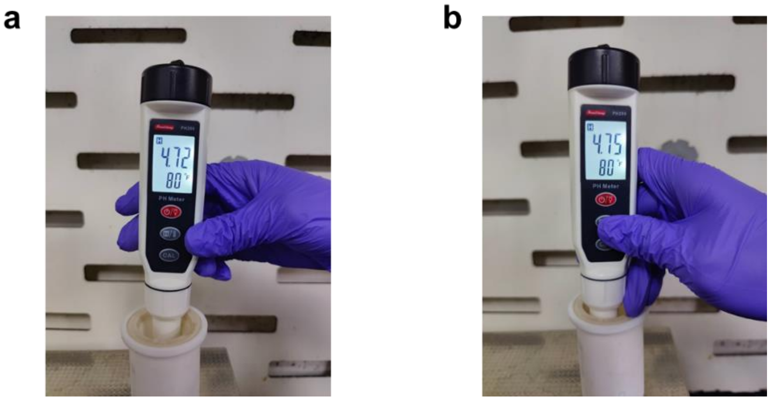

Figure S9. pH of precursor solutions **a** before and **b** after the addition of MEA.

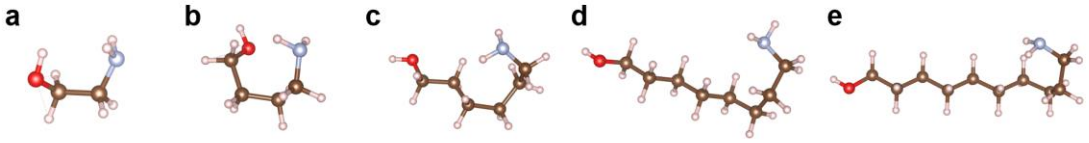

Figure S10. Optimized structures of a series of ethanolamine-based additives with varying alkyl chain lengths ($HO(CH_2)_nNH_2$, n=2, 4, 6, 8, and 10)

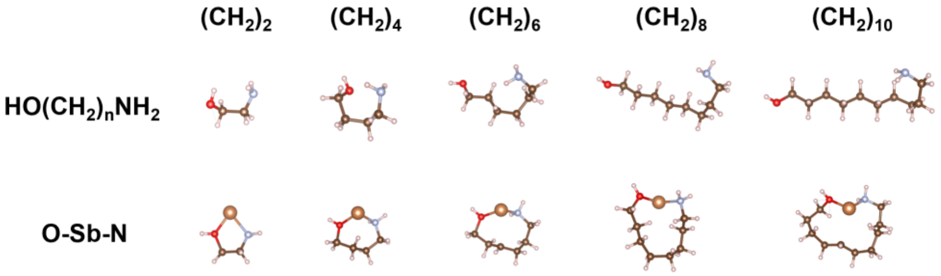

Figure S11. The coordination structures of $[Sb\text{-}HO(CH_2)_nNH_2]^{3+}$ (n=2, 4, 6, 8, and 10) .

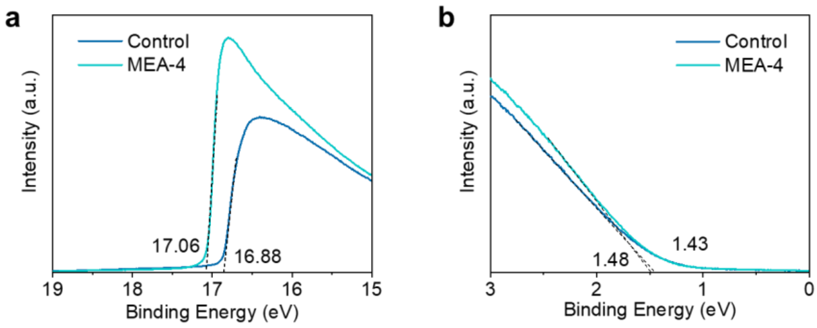

Figure S12. UPS spectra of the control and MEA-4 Sb_2S_3 film samples at the **a** secondary electron cut-off and **b** near the Fermi edge.

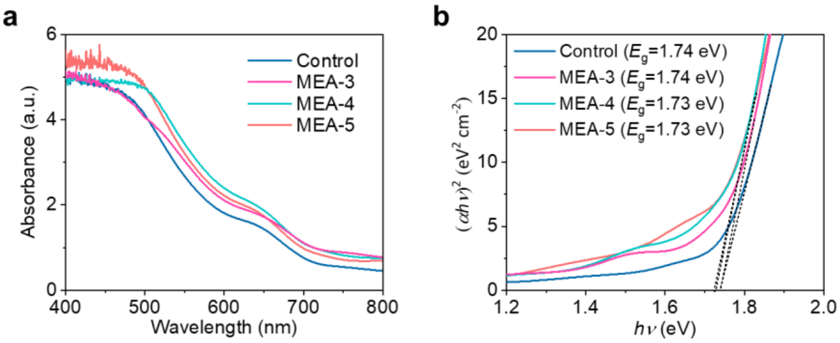

Figure S13. a UV–vis absorption spectra and corresponding **b** Tauc plots of the control and MEA-4 Sb_2S_3 films obtained with the addition of different MEA concentration. The bandgaps of Sb_2S_3 films are determined by the Tauc plots of UV-vis-NIR absorption spectra. The bandgap values of the control, MEA-3, MEA-4 and MEA-5 Sb_2S_3 films are estimated to be 1.74, 1.74, 1.73 and 1.73 eV, respectively. That is, there are no significant changes in the bandgap of Sb_2S_3 films with the addition of MEA.

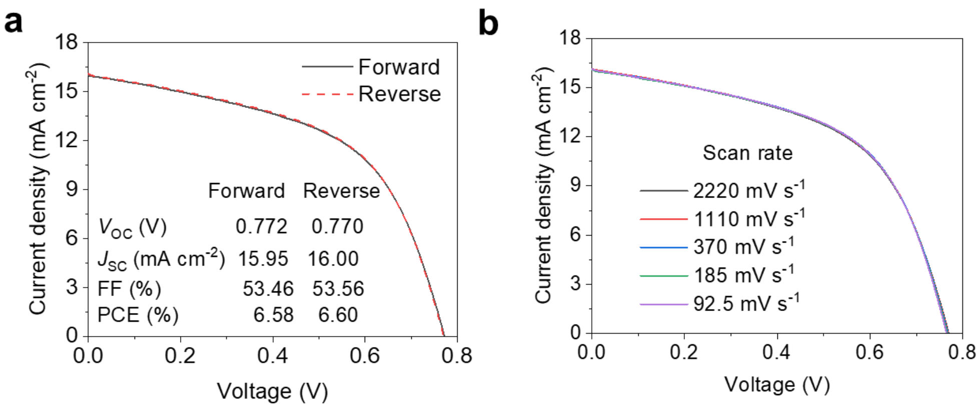

Figure S14. *J-V* curves of one of as-obtained planar Sb_2S_3 solar cells measured under **a** forward and reverse directions and **b** different scanning rates. The device is arbitrarily chosen to check the hysteresis of Sb_2S_3 solar cells.

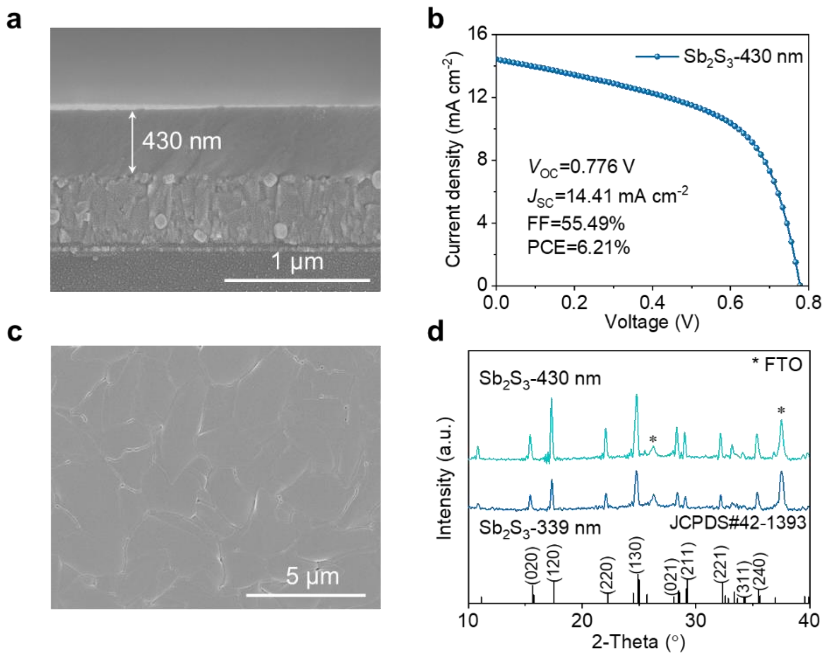

Figure S15. **a**, **c** Cross-sectional and top-view SEM images and **d** XRD patterns of the 480 nm-thick Sb_2S_3 film prepared by the traditional hydrothermal method. **b** *J-V* curve of Sb_2S_3 solar cell based on 480 nm-thick absorber films prepared by the traditional hydrothermal method.

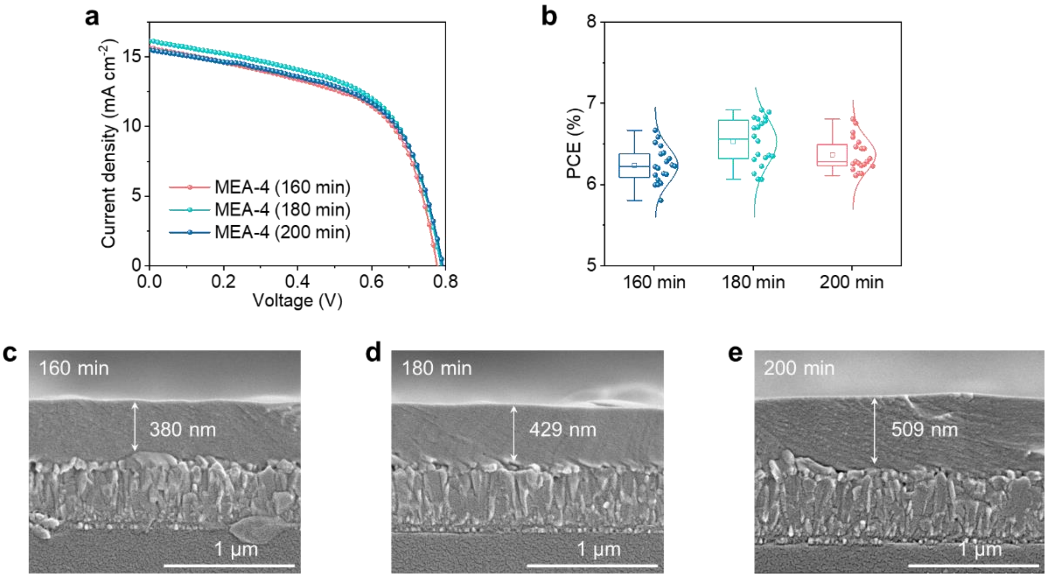

Figure S16. a *J-V* curves and **b** the statistics of PCEs for the MEA-4 Sb_2S_3 solar cells obtained under different hydrothermal durations, measured under AM 1.5G (100 mW cm^{-2}) illumination. **c-e** Cross-sectional SEM images of the MEA-4 Sb_2S_3 films obtained under the different hydrothermal durations of **c** 160 min, **d** 180 min and **e** 200 min.

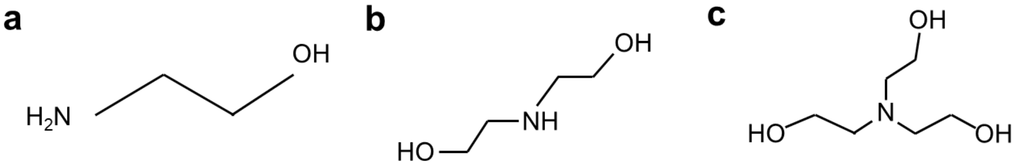

Figure S17. The molecular structures of **a** MEA, **b** DEA, and **c** TEA.

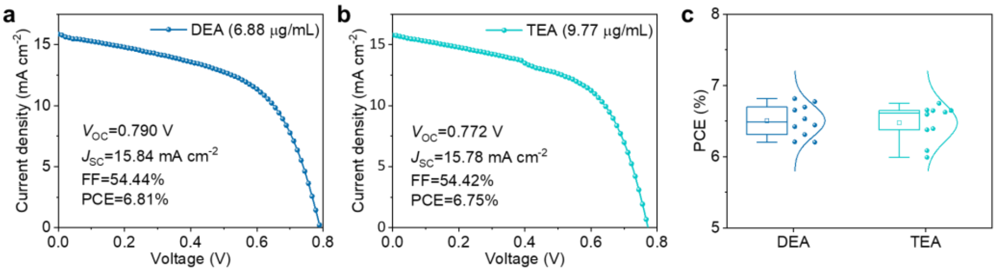

Figure S18. a, **b** *J-V* curves of Sb_2S_3 solar cells obtained with the addition of DEA or TEA, measured under AM 1.5G (100 mW cm^{-2}) illumination. **c** The statistics of PCEs for Sb_2S_3 solar cells obtained with the addition of DEA and TEA.

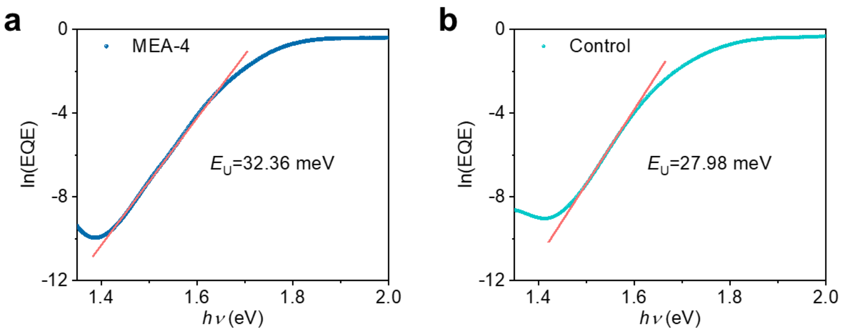

Figure S19. E_U values derived from the EQE spectra for **a** control and **b** MEA-4 Sb_2S_3 solar cells.

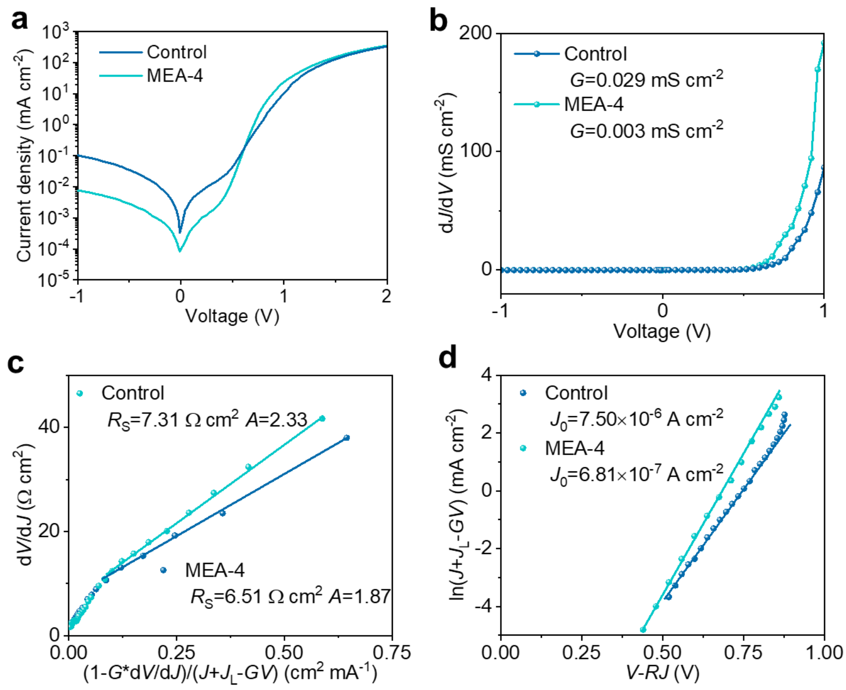

Figure S20. a The J-V curves measured under dark for the control and MEA-4 Sb_2S_3 solar cells, and corresponding fitting results of **b** the shunt conductance G, **c** dV/dJ versus (1-G*dV/dJ)/(J+J_L-GV) relationship curves for determining R and A, and **d** ln(J+J_L-GV) versus V-R_SJ relationship curves employed to determine J_0.

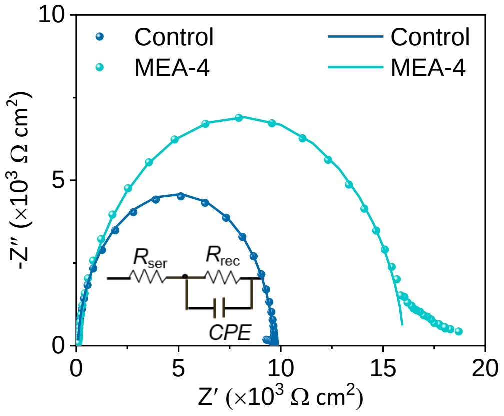

Figure S21. Nyquist plots of impedance spectra of the control and MEA-4 Sb_2S_3 solar cells, measured under dark at the bias voltage of 0.7 V. The equivalent circuit diagram given in the inset was used to perform the curve fitting.

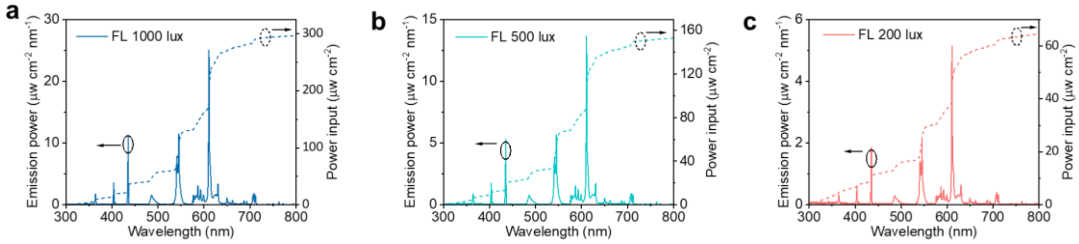

Figure S22. Spectral irradiance and integrated power densities of a 2700 K FL at **a** 1000, **b** 500, and **c** 200 lux.

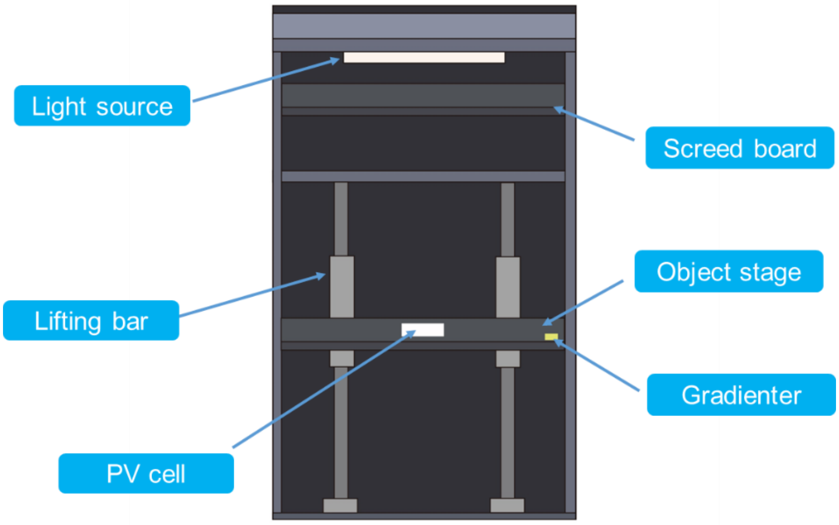

Figure S23. Schematic diagram of the black cabinet used for IPV measurements.

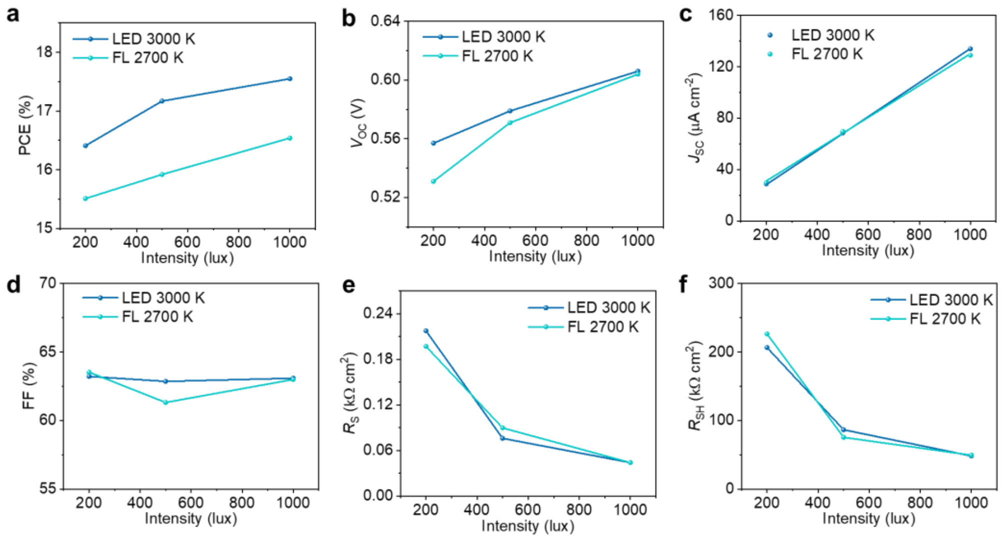

Figure S24. The dependences of **a** PCE, **b** V_{OC}, **c** J_{SC}, **d** FF, **e** R_S, **f** R_{SH} on the light intensity for MEA-4 Sb_2S_3 devices.

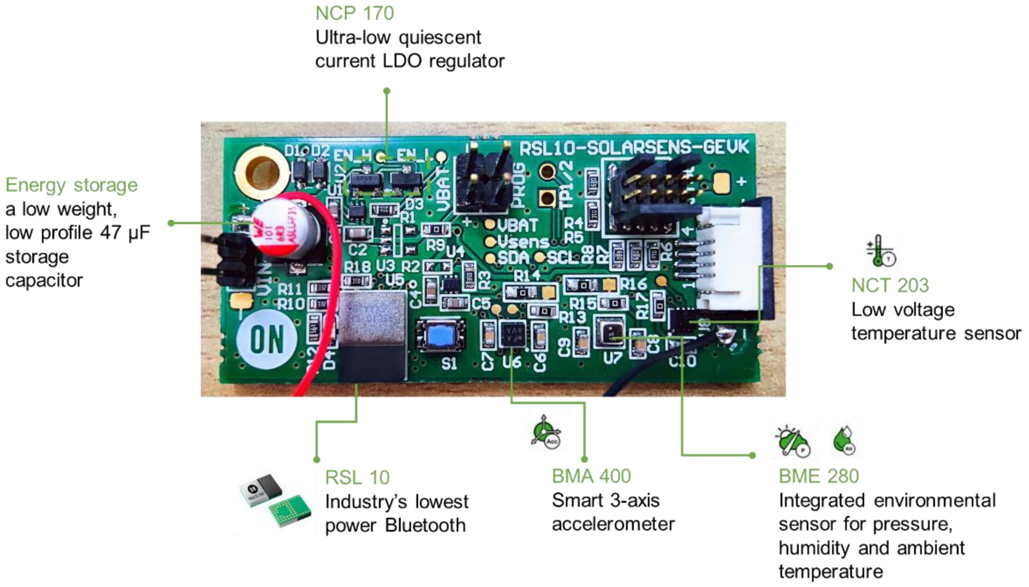

Figure S25. The picture of RSL10-SOLARSENS-GEVK, which is a comprehensive development platform for battery-less IoT applications in the smart building, smart home, and industrial sectors. The board is based on the industry's lowest-power Bluetooth® low-power radio (RSL10) and features sensors for environmental and motion sensing: the BMA400 smart 3-axis accelerometer, the BME280 smart environmental sensor and the NCT203 wide-range digital temperature sensor. The platform also features a lightweight, low-profile 47 F storage capacitor, a programming and

debugging interface, and an interface to a solar cell. Because the device harvests energy from a low current source, it is important to minimize leakage throughout the system during operation and standby. Along with other energy efficient devices, the platform's ultra-low quiescent current LDO (NCP170) significantly reduces leakage.

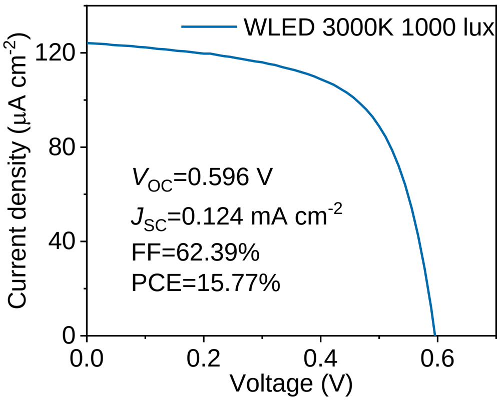

Figure S26. *J-V* curve of 1 cm^2 large-area MEA-4 Sb_2S_3 solar cells.

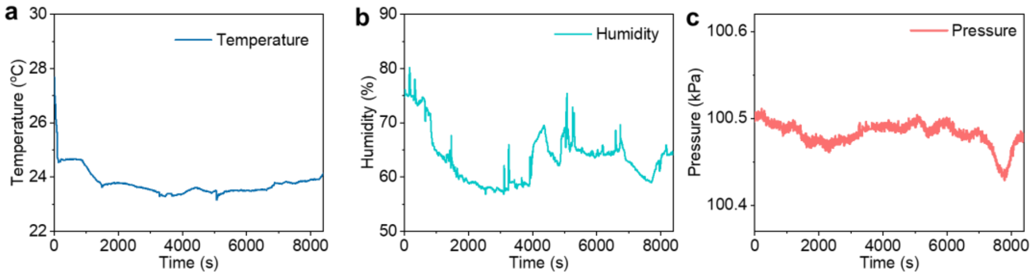

Figure S27. **a** Temperature, **b** humidity, and **c** atmospheric pressure variations recorded in the laboratory by the Sb_2S_3 module-driven sensor platform running continuously under 1000 lux LED light for 140 min

Table S1. Total energies of the species for the calculation of the formation energies* (per complex) of $[SbMEA]^{3+}$ for three different coordination scenarios as shown in Figure S6

Compound	Coordination atom	Energy (eV)
	N atom and O atom	-177536.7178
$[SbMEA]^{3+}$	Only N atom	-177535.5449
	Only O atom	-177536.2795

*Note: The formation energy of $[SbMEA]^{3+}$ is expressed as follows:

$$\Delta E_{\text{formation\_[SbMEA]}^{3+}} = E_{\text{[SbMEA]}^{3+}} - E_{[\text{Sb}(\text{H}_2\text{O})_3]^{3+}} - E_{\text{MEA}} + 3E_{\text{H}_2\text{O}}$$

where $E_{[\text{Sb}MEA]^{3+}}$ represents the energy of the complex, $E_{[\text{Sb}(\text{H}_2\text{O})_3]^{3+}}$, E_{MEA} and $E_{\text{H}_2\text{O}}$ denote the total energies of $[Sb(H_2O)_3]^{3+}$, MEA and H_2O, respectively. For the three different coordination scenarios, the total energies of $[Sb(H_2O)_3]^{3+}$, MEA and H_2O remain constant. Consequently, the formation energy is determined by the energy of $[SbMEA]^{3+}$. A lower energy of $[SbMEA]^{3+}$ corresponds to a lower formation energy, indicating a more stable complex.

Table S2. Total energies of the species for the calculation of the formation energies (per complex) of $[Sb(MEA)_n]^{3+}$

Compound	Energy (eV)
H_2O	-2078.369979
MEA	-5720.346618
$[Sb(H_2O)_3]^{3+}$	-178052.4758
$[SbMEA]^{3+}$	-177536.7178
$[Sb(MEA)_2]^{3+}$	-183264.3837
$[Sb(MEA)_3]^{3+}$	-188988.7669

*Note: In our study, we hypothesized the presence of three H_2O molecules complexed with Sb^{3+} based on the following considerations: (1) DFT calculations suggest that the replacement of water molecules by MEA in the hydration shell of Sb^{3+} leads to the formation of stable $[SbMEA]^{3+}$ complexes. These complexes exhibit lower formation energies when Sb^{3+} is coordinated with three H_2O molecules compared to more extensively hydrated Sb^{3+}. The total energies for the calculation of the formation energies of $[Sb(MEA)_n]^{3+}$: $[Sb(H_2O)_3]^{3+}$, -178052.4758 eV; $[Sb(H_2O)_4]^{3+}$, -

180133.6511 eV; $[Sb(H_2O)_5]^{3+}$, -182214.4069 eV. The formation energies of $[Sb(MEA)_n]^{3+}$ can be calculated by using Equation S20. (2) The steric hindrance around the Sb^{3+} can limit the number of H_2O molecules that can effectively coordinate to the cation. Three H_2O molecules might provide a reasonable balance between coordination and steric hindrance, ensuring stability and effective interaction within the complex. As MEA coordinates to Sb^{3+}, it replaces the H_2O molecules, potentially leading to complexes where multiple MEA molecules are coordinated.

Table S3. Total energies of $[Sb(MEA)_n]^{3+}$ (per complex) and MEA (per molecule) during the dissociation of $[Sb(MEA)_n]^{3+}$ into $[SbMEA]^{3+}$

Compound	Energy (eV)
$[Sb(MEA)_3]^{3+}$	-178.6428418
$[Sb(MEA)_2]^{3+}$	-118.9919737
$[SbMEA]^{3+}$	-59.40166542
MEA	-58.64758845

Table S4. Total energies during the process of $[SbMEA]^{3+}$ (per complex) binding to CdS substrate and MEA dissociation (per complex)

State	Energy (eV)
* + $[SbMEA]^{3+}$	-274.4810677
*$[SbMEA]^{3+}$	-276.2883443
*Sb^{3+} + MEA	-275.4489633

Table S5. Total energies of ethanolamine-based additives with varying alkyl chain lengths shown in Figure S10

Compound	Energy (eV)
$HO(CH_2)_2NH_2$	-5720.346618
$HO(CH_2)_4NH_2$	-7857.548115
$HO(CH_2)_6NH_2$	-9994.840598
$HO(CH_2)_8NH_2$	-12132.12542
$HO(CH_2)_{10}NH_2$	-14269.42818

*Note: According to the results from MEA, the simultaneous coordination of O and N atoms of $HO(CH_2)_nNH_2$ to Sb^{3+} were analyzed.

Table S6. Total energies of a series of Sb^{3+} complex with ethanolamine-based additives for different alkyl chain lengths ($HO(CH_2)_nNH_2$, n = 2, 4, 6, 8, and 10)

	Compound	Energy (eV)
$HO(CH_2)_2NH_2$	O-Sb-N	-177536.7178
$HO(CH_2)_4NH_2$	O-Sb-N	-179677.3441
$HO(CH_2)_6NH_2$	O-Sb-N	-181815.0086
$HO(CH_2)_8NH_2$	O-Sb-N	-183952.6725
$HO(CH_2)_{10}NH_2$	O-Sb-N	-186091.6441

Table S7. Formation energies of a series of Sb^{3+} complex with ethanolamine-based additives for different alkyl chain lengths ($[Sb\text{-}HO(CH_2)_nNH_2]^{3+}$, n=2, 4, 6, 8, and 10)

	Compound	Formation energy (eV)
$[SbMEA]^{3+}$	O-Sb-N	0.9947
$[Sb\text{-}HO(CH_2)_4NH_2]^{3+}$	O-Sb-N	-2.4301
$[Sb\text{-}HO(CH_2)_6NH_2]^{3+}$	O-Sb-N	-2.8021
$[Sb\text{-}HO(CH_2)_8NH_2]^{3+}$	O-Sb-N	-3.1812
$[Sb\text{-}HO(CH_2)_{10}NH_2]^{3+}$	O-Sb-N	-4.8500

*Note: The results demonstrate that, as the alkyl chain length increases, the additives become more favorable for complexing with hydrated Sb^{3+} in the solution.

Table S8. Band structures of the control and MEA-4 Sb_2S_3 thin films

Samples	E_{onset}	E_{cutoff}	E_F	VBM	CBM
Control	1.43	16.88	-4.34	-5.77	-4.03
MEA-4	1.48	17.06	-4.16	-5.64	-3.91

Table S9. Photovoltaic performance parameters of the MEA-4 Sb_2S_3 solar cells obtained under different hydrothermal durations, measured under AM 1.5G (100 mW cm^{-2}) illumination

Device	V_{OC} (V)	J_{SC} (mA cm^{-2})	FF (%)	PCE (%)
MEA-4 (160min)	0.776	15.63	54.02	6.89
MEA-4 (180min)	0.787	16.12	56.92	7.22
MEA-4 (200min)	0.784	15.58	56.57	6.91

Table S10. The device performance parameters of junction ideality factor (A), the reverse saturation current density (J_0), the series resistance (R_s), and the shunt conductance (G, i.e., $1/R_{sh}$) extracted from J-V curves of the control and MEA-4 Sb_2S_3 solar cells measured under dark condition

Device	G (mS cm^{-2})	R_S (Ω cm^2)	A	J_0 (mA cm^{-2})
Control	0.029	7.31	2.33	7.50×10^{-6}
MEA-4	0.003	6.51	1.87	6.81×10^{-7}

Table S11. The fitting results for impedance spectra of the control and MEA-4 Sb_2S_3 solar cells by using the Z-view software, measured under dark at the bias voltage of 0.7 V

Device	R_{ser} (Ω cm^2)	R_{rec} (Ω cm^2)	CPE-T (F)	CPE-P (F)
Control	4.92	9615	2.26×10^{-9}	0.96
MEA-4	3.78	16020	5.76×10^{-9}	0.92

Table S12. The fitting results of TA spectra for the control and MEA-4 Sb_2S_3 films. The lifetime τ_{av} can be calculated using the equation $\tau_{av} = (A_1\tau_1^2 + A_2\tau_2^2)/(A_1\tau_1 + A_2\tau_2)$

Device	A_1	τ_1 (ps)	A_2	τ_2 (ns)	τ_{av} (ns)
Control	0.490	536.0	0.510	5.35	4.93
MEA-4	0.484	572.0	0.516	7.07	6.61

Table S13. The input power values calculated from the power density spectra of light sources

Light source	1000 lux (μW cm^{-2})	500 lux (μW cm^{-2})	200 lux (μW cm^{-2})
3000K LED	291.9	145.2	62.0
2700K FL	296.7	153.1	64.6

Table S14. Performance of perovskite-inspired materials for IPV. Please note that none of the devices from different groups were measured under identical conditions. However, it is useful to tabulate the power conversion efficiencies (PCEs) reported to gauge the current status of the field

Year	Light-absorber	Device structure	PCE (%)	Ref
2021	BiOI	ITO/NiO_x/BiOI/ZnO/Cr/Ag	4.0	[14]
2021	$Cs_3Sb_2Cl_xI_{9-x}$	FTO/c-TiO_2/m-TiO_2/$Cs_3Sb_2Cl_xI_{9-x}$/ poly-TPD/Au	4.4	[14]
2022	$(Cs,MA,FA)_3Sb_2(I,Cl)_9$	FTO/c-TiO_2/ $(Cs,MA,FA)_3Sb_2(I,Cl)_9$/P3HT/Au	6.37	[15]
2021	$AgBiI_4$	FTO/c-TiO_2/$AgBiI_4$/Spiro-OMeTAD/Au	5.17	[16]
2023	Ag_2BiI_5	ITO/SnO_2/ Ag_2BiI_5/PTAA/Au	5.0	[17]
2022	Cu_2AgBiI_6	FTO/c-TiO_2/m-TiO_2/Cu_2AgBiI_6/Spiro-OMeTAD/Au	5.52	[18]
2023	$Cu_2Ag(Bi,Sb)I_6$	FTO/c-TiO_2/m-TiO_2/ $Cu_2Ag(Bi,Sb)I_6$/Spiro-OMeTAD/Au	9.53	[19]
2022	Se	FTO/c-TiO_2/Te/Se/Au	15.1	[20]
2022	Sb_2S_3	FTO/SnO_2/CdS/Sb_2S_3/Spiro-OMeTAD/Au	16.37	[21]
2024	Sb_2S_3	FTO/SnO_2/CdS/Sb_2S_3/Spiro-OMeTAD/Au	17.55	This work

References

1 Wang, S. *et al.* A Novel Multi-Sulfur Source Collaborative Chemical Bath Deposition Technology Enables 8%-Efficiency Sb_2S_3 Planar Solar Cells. *Advanced Materials* **34**, 2206242 (2022).

2 Zhang, L. *et al.* Mechanistic Study of the Transition from Antimony Oxide to Antimony Sulfide in the Hydrothermal Process to Obtain Highly Efficient Solar Cells. *ChemSusChem* **16**, e202202049 (2023).

3 Hegedus, S. S. & Shafarman, W. N. Thin-film solar cells: device measurements and analysis. *Progress in Photovoltaics: Research and Applications* **12**, 155-176 (2004).

4 Shen, K. *et al.* CdTe solar cell performance under low-intensity light irradiance. *Solar Energy Materials Solar Cells* **144**, 472-480 (2016).

5 Cui, Y. *et al.* Wide-gap non-fullerene acceptor enabling high-performance organic photovoltaic cells for indoor applications. *Nature Energy* **4**, 768-775 (2019).

6 Green, M. A. Solar cell fill factors General graph and empirical expressions. *Solid-State Electronics* **24**, 788-789 (1981).

7 Chen, C. H. *et al.* Full-Dimensional Grain Boundary Stress Release for Flexible Perovskite Indoor Photovoltaics. *Advanced Materials* **34**, e2200320 (2022).

8 Tang, R. *et al.* n-Type Doping of Sb_2S_3 Light-Harvesting Films Enabling High-Efficiency Planar Heterojunction Solar Cells. *ACS Applied Materials & Interfaces* **10**, 30314-30321 (2018).

9 Vinayakumar, V. *et al.* Effects of rapid thermal processing on chemically deposited antimony sulfide thin films. *Materials Science in Semiconductor Processing* **80**, 9-17 (2018).

10 Zhou, H. *et al.* Effective additive for enhancing the performance of Sb_2S_3 planar thin film solar cells. *Journal of Materiomics* **7**, 1074-1082 (2021).

11 Kusumi, T. *et al.* Mechanism of Light-Soaking Effect in Inverted Polymer Solar Cells with Open-Circuit Voltage Increase. *ACS Omega* **2**, 1617-1624 (2017).

12 Panghulan, G. R. *et al.* Synthesis of TiN/N-doped TiO_2 composite films as visible light active photocatalyst. *Journal of Vacuum Science & Technology B* **38**, 062203 (2020).

13 Han, J. *et al.* Multidentate anchoring through additive engineering for highly efficient Sb_2S_3 planar thin film solar cells. *Journal of Materials Science & Technology* **89**, 36-44 (2021).

14 Peng, Y. H. *et al.* Lead-Free Perovskite-Inspired Absorbers for Indoor Photovoltaics. *Advanced Energy Materials* **11**, 2002761 (2021).

15 Lamminen, N. *et al.* Triple A-Site Cation Mixing in 2D Perovskite-Inspired Antimony Halide Absorbers for Efficient Indoor Photovoltaics. *Advanced Energy Materials* **13**, 2203175 (2023).

16 Turkevych, I. *et al.* Potential of $AgBiI_4$ rudorffites for indoor photovoltaic energy harvesters in autonomous environmental nanosensors. *Japanese Journal of Applied Physics* **60**, SCCE06 (2021).

17 Guerrero, N. B. C. *et al.* A Semitransparent Silver-Bismuth Iodide Solar Cell with Voc above 0.8 V for Indoor Photovoltaics. *ACS Applied Energy Materials* **6**, 10274-10284 (2023).

18 Grandhi, G. K. *et al.* Perovskite-inspired Cu_2AgBiI_6 for mesoscopic indoor photovoltaics under realistic low-light intensity conditions. *Sustainable Energy & Fuels* **7**, 66-73 (2022).

19 Al-Anesi, B. *et al.* Antimony-Bismuth Alloying: The Key to a Major Boost in the Efficiency of Lead-Free Perovskite-Inspired Photovoltaics. *Small* **19**, 202303575 (2023).

20 Yan, B. *et al.* Indoor photovoltaics awaken the world's first solar cells. *Science Advances* **8**, eadc9923 (2022).

21 Zheng, J. Z. *et al.* Enhanced hydrothermal heterogeneous deposition with surfactant additives for efficient Sb_2S_3 solar cells. *Chemical Engineering Journal* **446**, 136474 (2022).
